# Self-immolative polydisulfides and their use as nanoparticles for drug delivery systems†

**DOI:** 10.1039/d4ra07228f

**Published:** 2024-11-07

**Authors:** Katharina Völlmecke, Maurice Kramer, Corinna Horky, Oliver Dückmann, Dennis Mulac, Klaus Langer, Dirk Kuckling

**Affiliations:** a Paderborn University Warburger Straße 100 33098 Paderborn Germany dirk.kuckling@uni-paderborn.de; b Institute of Pharmaceutical Technology and Biopharmacy, University of Münster Corrensstr. 48 D-48149 Münster Germany

## Abstract

Over the last few decades, nanotechnology has established to be a promising field in medicine. A remaining dominant challenge in today's pharmacotherapy is the limited selectivity of active pharmaceutical ingredients and associated undesirable side effects. Controlled drug release can be promoted by smart drug delivery systems, which release embedded API primarily depending on specific stimuli. Consequently, also the microenvironment of tumor tissue can be used advantageously. Dithiothreitol (DTT) based self-immolative polydisulfides were synthesized that preferentially respond to pathologically increased glutathione (GSH) concentrations, as found in solid tumors. The synthesis with different degrees of polymerisation was investigated as well as the synthesis of a copolymer consisting of dithiothreitol and butanedithiol (BDT). Toxicity tests were carried out on pure polymers and their degradation products. The ability to degrade was examined at pathological and physiological glutathione concentrations in order to test the suitability of the polymer as a matrix for nanoparticulate carrier systems. In addition, the processability of one polymer into nanoparticles was investigated as well as the degradation behaviour with glutathione.

## Abstract 2

Polydisulfide based nanoparticles can be degraded by glutathione (GSH) to deliver hydrophobic drugs.

## Graphical abstract

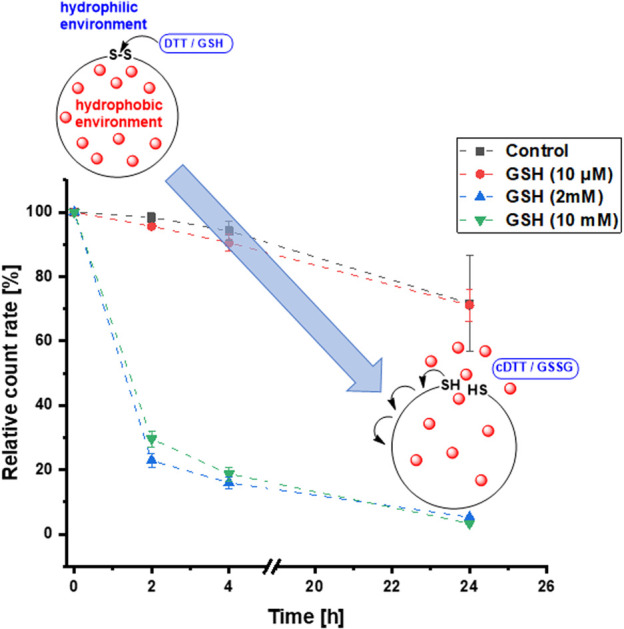

## Introduction

The American Cancer Society found that 1 out of 8 women will be diagnosed with invasive breast cancer and 3% of all women will die of it, making it the most prevalent type of cancer globally.^1,2^ The numbers of diagnoses are estimated to rise to 3 million cases per year with an estimated one million deaths by 2040.^3^ The increasing importance of cancer treatment therefore leads to the development of a multitude of different drug delivery systems, which aim to improve the efficacy and reduce the side effects of common cytostatic drugs.^4,5^ Higher efficacy implies that lower doses are needed and thereby side effects can be reduced. The efficacy can be increased by prolonging the circulation time, by preventing the metabolisation or premature clearance of the theranostic, or by enhancing the hydrophilicity of the mostly hydrophobic drugs.^6^ Another way to reduce side effects is by making use of active (*e.g.* by antibodies, glycoproteins, peptides) or passive targeting.^7^ Passive targeting is possible due to the enhanced permeability and retention (EPR) effect which allows colloidal particles to penetrate tumor tissue. While there is a lot of research on drug delivery systems, very few make the clinical translation.^8^ Some of the challenges include poor stability, potential toxicity, transport across biological barriers and difficulty in scale-up. Some of them can be overcome by using self-immolative polydisulfides as a base for polymeric nanoparticles. Polydisulfides have emerged as versatile compounds *e.g.* as organic cathodes for batteries^9^ or as self-healing materials due to their dynamic covalent bonds.^10,11^ Another growing field is the use of polydisulfides in biocompatible materials and nanomedicine.^12–14^ Many tumors and breast cancer in particular were shown to have elevated levels of reductive tripeptide glutathione (GSH).^15,16^ Therefore, their structure allows the polydisulfides to be used as stimuli-responsive carriers. Another advantage that is inherent to polydisulfides, is the possibility to escape the endosomal route by thiol-mediated uptake.^17^ One of the most prominent examples of polydisulfides used in drug delivery systems is poly(lipoic acid)^18–20^ due to its makeup of endogenous building blocks. Other examples of degradable polydisulfides are a 1,6-hexanedithiol-based copolymers used in self-assembled nanocarriers, showing that they are a promising platform for drug delivery applications.^21–23^ While self-healing behaviour of polymeric capsules may be wanted before reaching the target site, it can be obstructive for burst release mechanisms. Self-immolative polymers can overcome this obstacle due to their unique degradation mechanism: an end-cap cleavage will trigger a degradation cascade of the polymer backbone, which will leave only monomeric units.^24^ Triggers include light irradiation (UV to NIR),^25,26^ redox reactions,^21,27^ temperature changes^28^ or changes of pH.^29^ Well known examples of polymers showing self-immolative behaviour are polyphthalaldehydes,^25,30^ poly(benzyl ethers)^31^ and polycarbamates^32^ or polycarbonates.^33^ Self-immolative structures have already found application in nanomedicine *e.g.* as prodrug spacers that can release the bound molecule upon the specific trigger.^34^ Other self-immolative polymers have been used as micelles, microcapsules,^32^ polymersomes^26^ or gate keepers^29^ that can encompass larger quantities of drug molecules. Oftentimes drug delivery systems suffer from high production costs and scalability during clinical translation.^8^ However, in this work homo- and copolymers of non-toxic dithiothreitol (DTT) and 1,4-butanedithiol (BDT) are used to form nanoparticles using inexpensive, facile and scalable techniques. Since Hansen-Felby *et al.* did report the degradation of 1,6-butanedithiol based polymers to undergo unspecific degradation leading to larger macrocycles, this report will focus on structures that avoid larger degradation fragments.^35^ Moreover, the degradation speed was shown to be increased by the formation of stable degradation products such as six-membered rings. The hydrophobicity of the polymers was tuned by the synthesis of different copolymers, as a more hydrophobic backbone should improve the encapsulation of hydrophobic drugs.

## Materials and methods

### Materials

All materials were used without further purification. *N*,*N*-Dimethylacetamide (DMAc) (99.5%), *N*,*N*-dimethylformamide (DMF) (anhydrous, amine free, 99.9%), dithiothreitol (DTT, electrophoresis, 99%), lithium bromide (anhydrous, 99+%), dimethylsulfoxid (DMSO) (≥99.9%) as well as tris(hydroxymethyl)aminomethane (≥99%) (TRIS), 2,6-di-*tert*-butyl-4-methylphenol (BHT) (99%) (Fluka), l-glutathione, reduced (GSH), ≥98% used for the particle degradation studies and l-glutathione reduced (97%) (Alfa Aesar) used for GPC and NMR studies were purchased from Fisher Chemical (Thermo Fisher Scientific Inc., Germany). Dimethylsulfoxide-d_6_ (DMSO-d_6_) (99.8%) was obtained from Deutero GmbH (Kastellaun, Germany). 2,2′-Dipyridyl disulfide (DPDS) (98%) and Nile red (NR) were purchased from abcr (Karlsruhe, Germany). Tetrahydrofuran (THF) (p.A.) was purchased from Stockmeier (Bielefeld, Germany). Polyvinyl alcohol (PVA) and Isopore™ PC membranes were purchased from Merck KGaA (Darmstadt, Germany). SK-BR-3 cells were obtained from ATCC® (LGC Standards GmbH, Wesel, Germany) and the cell line MCF-7 was kindly provided by Dr Spänkuch (Universitäts-Frauenklinik, Tübingen). All used cell culture materials for cell cultivation and cell proliferation reagent WST-1 were purchased from Sigma-Aldrich (Steinheim, Germany).

### Syntheses

#### General procedure of synthesis of pDTT

Detailed synthetic procedures of the polymers can be found in the ESI.† DTT was placed in Schlenk flask under protective atmosphere. DPDS was added and it was evacuated and flushed with nitrogen again. The solids were stirred and the powder turned from white to yellow. 2.7 mL of anhydrous DMF were added and stirred at room temperature. After 17 h the solution was turbid, 1.5 mL DMF were added to resolve the precipitate. After 2 min the yellow solution was precipitated from 1 : 1 chloroform/isohexane mixture and centrifuged for 15 min. The supernatant was removed and a sample of the solid was taken for NMR analysis. The residue was dissolved in DMF. The precipitation was repeated three times to give a light yellow solid that was dried *in vacuo* overnight.

#### General procedure of synthesis of p(DTT-*co*-BDT)

Detailed synthetic procedures of the polymers can be found in the ESI.† BDT was placed in a Schlenk flask under protective atmosphere. DTT was added. The solids were stirred and the powder turned from white to yellow. The mixture was cooled in an ice bath for 5 min. 2.7 mL of dry DMF were added and stirred at 0 °C. After one hour the solution became turbid. After 24 h the suspension was diluted with 0.5 mL DMF and precipitated from 1 : 1 chloroform/isohexane mixture and centrifuged for 15 min. The residue was resolved in a DMF–THF mixture and precipitated again. The precipitation was repeated four times to give a yellow solid that was dried *in vacuo* overnight.

### Gel permeation chromatography

The molecular weight and molecular weight distribution were determined in dimethylacetamide and 0.5 g L^−1^ LiBr with BHT used as an internal standard at a flow rate of 1 mL min^−1^. The system was equipped with a precolumn PSS-GRAM 10 μm, consecutive columns PSS-GRAM 10^2^ Å, PSS-GRAM 10^3^ Å, and PSS-GRAM 10^4^ Å, an RI detector (Waters RI 2410), a UV-vis detector (Merck PDA-L3000), and a viscosity detector (PSS ETA 2010). Samples were prepared with a concentration of 6 mg mL^−1^ if not stated otherwise. Molar masses were obtained according to the calibration using polystyrene standards. The molar mass and molar mass distribution were determined in dimethylacetamide containing 0.5 g L^−1^ LiBr and 4 g L^−1^ BHT used as an internal standard. Signals of UV-vis, RI and viscosity detectors were used for the analysis. To study the effect of 2 μM GSH concentration the following procedure was used: approximately 30 mg polymer were dissolved in 5 mL GPC solvent. A stock solution of glutathione in the same solvent was prepared (*ca.* 1.63 mmol L^−1^) and 6.2 μL were added to the polymer solution (total concentration of glutathione should be 2 μM) under stirring at room temperature. A quenching solution was prepared from 2,2′-dipyridyl disulfide in the GPC solvent (0.02 mol L^−1^). Aliquots (0.9 mL) of the mixture were put into 5 centrifugation tubes which were then incubated at 37 °C in a ThermoMixer® stirring at 900 rpm to keep the glutathione suspended. After the respective times the reactions were quenched by addition of 0.1 mL quenching solution and stored refrigerated until the measurement. Before the analysis the solutions were filtered using 0.45 μm syringe filters. To study the effect of 2 mM and 10 mM concentration the procedure was adapted as follows: 7.4 μL of the GSH stock solution (*ca.* 0.15 mol L^−1^ for 2 mM; 0.66 mol L^−1^ for 10 mM) were added to the polymer solution (6 mg mL^−1^) under stirring at room temperature. It was quenched with DPDS solution (0.09 mol L^−1^ for 2 mM, 0.38 mol L^−1^ for 10 mM) and the experimental procedure was carried on as described above.

### Nuclear magnetic resonance spectroscopy

NMR spectroscopy was conducted on a Bruker Avance500 Spectrometer. ^1^H-NMR spectra were measured at 500 MHz and 30 °C. ^13^C and kinetic ^1^H measurements were conducted on a Bruker Ascent 700 spectrometer at 700 MHz and 30 °C. Measurements were conducted in DMSO-d_6_. Spectra analysis was carried out using TopSpin 4.3.0 and ACD Spectrus Processor. For kinetic measurements approximately 20 mg of polymer were dissolved in 0.6 mL DMSO-d_6_ and analysed by ^1^H-NMR. A stock solution of glutathione in DMSO-d_6_ was prepared (0.53–0.74 mol L^−1^). Two equivalents GSH were added to the polymer solution from the GSH stock solution. Further details can be found in the ESI.†^1^H-NMR was recorded immediately after addition and then hourly for up to 16 h.

### Nanoparticle preparation and analysis

#### Nanoparticle preparation

The polymer pDTT_36_ was processed into nanoparticles using the well-established solvent displacement method. Briefly, 5 mg pDTT_36_ were dissolved in 0.5 mL DMSO. The organic polymer solution was injected into 5 mL of a stirring, aqueous solution of 2 wt% PVA (previously filtered). The suspension was stirred overnight at 550 rpm. The suspension was purified by centrifugation (15 000 g, 60 min, 4 °C) and redispersion in ultrapure water.

#### Nanoparticle characterization

The hydrodynamic diameter and polydispersity index were determined in triples by dynamic light scattering (DLS) using a Malvern Zetasizer Nano ZS (Malvern Instruments Ltd, Malvern, UK). Therefore, aliquots of 20 μL aqueous nanoparticle suspension were diluted with 1 mL of ultrapure water and measured at 22 °C at a backscattering angle of 173°. Afterwards, the system was switched to zeta mode (applied electrical voltage of 140 V at 22 °C) to determine the zeta potential.

#### Visualization of nanoparticles

An aliquote of 3 μL aqueous suspension (0.25 mg mL^−1^) was applied to a Isopore™ PC membrane filter 0.1 μm and dried overnight. Afterwards the samples were sputtered with gold under argon atmosphere (Sputter SCD 040, BALTEC, Liechtenstein) and images were taken with a CamScan CS4 microscope (Cambridge Scanning Company, Cambridge, UK). Graphical analysis was done by Imagej.

#### Degradation trial

Lyophilized nanoparticles at a concentration of 0.1 mg mL^−1^ were incubated at 37 °C with different glutathione concentrations (10 μM, 2 mM and 10 mM) in TRIS buffer (0.1 M). Control samples were measured in TRIS buffer only. Degradation of nanoparticles was tracked by the progression of the DLS count rate.

#### Cell lines and cell culture

MCF-7 cells were cultivated at 10% CO_2_ atmosphere in high glucose DMEM supplemented with 10 vol% FBS, 1 vol% of a mixture containing penicillin (10 000 U mL^−1^) and streptomycin (10 mg mL^−1^), 1 vol% gentamicin solution (10 mg mL^−1^), 1 vol% MEM non-essential amino acid solution (100×) and 1 vol% l-glutamine solution (200 mM). SK-BR-3 cells were grown at 5% CO_2_ atmosphere in modified McCoy's 5A Medium supplemented with 20 vol% FBS, 1 vol% of a mixture containing penicillin (10 000 U mL^−1^) and streptomycin (10 mg mL^−1^), 1 vol% gentamicin solution (10 mg mL^−1^), 50 μL EGF and 1 vol% l-glutamine solution (200 mM). All cell lines were cultivated at 37 °C and split after reaching a confluency of about 80%. All studies were carried out within a maximum of 15 passages.

#### Cell viability by WST-1 assay

To investigate the cytotoxicity of pDTT_21_, pDTT_36_, pDTT_69_ and their degradation products, the WST-1 assay was used. For this, cells were seeded on 96 well plates in a concentration of 20 000 cells per well. After reaching a confluency of 80–90%, the culture medium was replaced by test media. For intact polymers, several dilutions of each pDTT stock solution (50 mg mL^−1^ in DMSO) were prepared in serum free DMEM as suspensions. For testing of degradation products, the polymer stock solutions were diluted with 10 mM GSH in serum free DMEM to a concentration of 500 μg mL^−1^. These suspensions were incubated in a thermomixer at 37 °C and 550 rpm for at least 4 h before cell incubation. Hereby, clear solutions were obtained. Degradation solutions were diluted with serum free DMEM to further test concentrations. After 24 h cell incubation, test media were removed and each well was washed with 100 μL serum free medium. Washing medium was substituted for 100 μL serum free medium per well. A blank measurement was taken at 460 nm in a BioTek Synergy Mx microplate reader and 10 μL cell proliferation reagent WST-1 were immediately added per well. For experiments with MCF-7 cells, the absorbance was recorded again after 45 min incubation at 37 °C. SK-BR-3 cells were remeasured after 3 h incubation. To report data as relative cell viability, a two-point calibration was carried out at each 96 well plate. Serum free medium was used as negative control corresponding to 100% viability and 20% DMSO in serum free medium as positive control corresponding to 0% viability. All experiments were conducted three times over three cell passages as technical triplicates and are presented as mean ± standard deviation.

#### Nile red loaded nanoparticle preparation and fluorescence emission analysis

11.4 mg pDTT_36_ were dissolved in 1.12 mL DMSO and 0.02 mL of a Nile red stock solution (1 mg mL^−1^) were added. Due to the low solubility of nile red the concentration must be kept low to prevent precipitation. The organic polymer solution was injected into 5 mL of a stirring, aqueous solution of 2 wt% PVA (previously filtered). The suspension was stirred overnight at 550 rpm. The suspension was purified by centrifugation (15 000 g, 60 min, 4 °C) and redispersion in ultrapure water. 0.2 mL particle suspension were diluted in 1.8 mL TRIS buffer and stored in the thermo shaker in between fluorescence and DLS measurements. DLS and fluorescence spectra were measured after 1 h, 2 h, 3 h, 4 h, and 16 h. Fluorescence emission analysis was conducted using a Spectrofluorometer 8300 by JASCO (*λ*_ex_ = 560 nm, *λ*_em_ = 600–800 nm).

#### Statistical analysis

Experiments were conducted in triplicate. Unless otherwise specified, results are presented as average values with standard deviation. Sigma Plot 12.5 (Systat Software GmbH, Erkrath, Germany) was used for analysis of variance (one way ANOVA followed by Holm-Sidak post-hoc tests). If significant, relations are marked with asterisk (* for *p* ≤ 0.05).

## Results and discussion

### Synthesis of homo- and copolymers

**Scheme 1:**
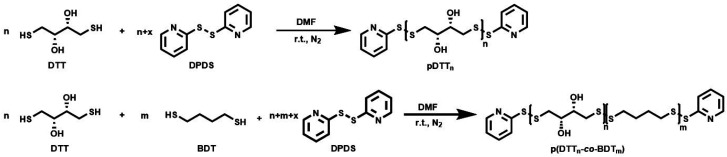
Reaction scheme of the synthesis of homopolymer pDTT and copolymer p(DTT_*n*_-*co*-BDT_*m*_).

The polymers were synthesized from commercially available materials following a modified procedure described by Basak *et al* (Scheme 1)*.*^36^ DTT and in case of copolymers the respective amount of BDT and a slight excess of DPDS (*x*) were placed in a schlenk flask under inert atmosphere (Table 1). Educt ratios of the comonomers used translated well into the incorporated monomer ratios. High concentrations are necessary to synthesize polydisulfides^37^ and Pal *et al.* even developed a solvent free method.^38^ To maintain a uniform method of synthesis for both homo- and copolymers, the educts were dissolved in a small amount of DMF. The degree of polymerisation (DP) can be tuned by the excess of DPDS, but using this procedure could not reach the theoretical DP that is calculated as follows:DP = 1/*x*

**Table 1:** Ratio of DPDS and BDT to DTT, *M*_n_ as calculated from NMR (d_6_-DMSO), *M*_n_ and polydispersity from GPC (DMAc + 0.5 g L^−1^ LiBr, reference: PS), yield, repeating units *P*_n,DTT_ and *P*_n,BDT_ from NMR analysis

	DPDS/DTT/BDT	Reaction temperature	*M*_n,GPC_ [g mol^−1^]	*Đ*_GPC_	*M*_n,NMR_ [g mol^−1^]	*P*_n,DTT_	*P*_n,BDT_	DTT content [%]	Yield [%]
pDTT_21_	1.03/1/—	r.t.	4800	1.7	3420	21	—	100	53
pDTT_36_	1.01/1/—	r.t.	5600	2.1	5700	36	—	100	56
pDTT_69_	1.01/1/—	0 °C	10 800	3.0	10 730	69	—	100	58
p(DTT_14_-*co*-BDT_9_)	1.70/1/0.66	0 °C-r.t.	3600	2.1	3430	14	9	60	22
p(DTT_16_-*co*-BDT_7_)	1.46/1/0.43	0 °C-r.t.	3700	2.3	3930	16	7	70	33
p(DTT_22_-*co*-BDT_3_)	1.28/1/0.25	0 °C-r.t.	3900	2.5	3500	22	3	88	40

However, polymers of higher molecular weight could be obtained by decreasing the reaction temperature, as the mixture heats up during the process. All polymers were purified by fourfold precipitation from a chloroform/iso-hexane mixture and obtained as a light yellow solid in yields of about 50%. DP and *M*_n_ were calculated from ^1^H NMR results according to the work of the Daasbjerg group.^35,38^*M*_n_ ranged between 3000 and 11 000 g mol^−1^ and molecular weights obtained by ^1^H NMR analysis were in good agreement with the GPC results (polystyrene reference). Moreover, GPC analysis revealed a unimodal distribution for all polymers with dispersities between 1.7–3, which is typical for polycondensation reactions.

### Polymer degradation

**Fig. 1:**
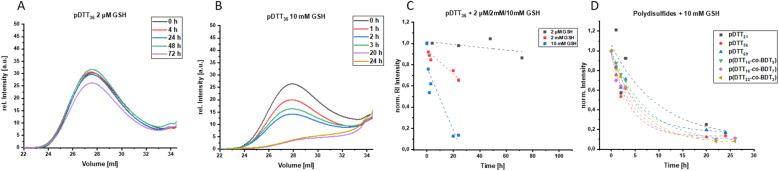
Glutathione induced degradation of homo- and copolymers; GPC elution curves of degradation of pDTT_36_ at *c*_GSH_ = 2 μM (A), GPC elution curves of degradation of pDTT_36_ at *c*_GSH_ = 10 mM (B), norm. intensities of pDTT_36_ from RI signal at peak maximum at *c*_GSH_ = 10 mM, *c*_GSH_ = 2 mM and *c*_GSH_ = 2 μM (C), norm. intensities of homo- and copolymers from RI signal at peak maximum at *c*_GSH_ = 10 mM (D).

**Fig. 2:**
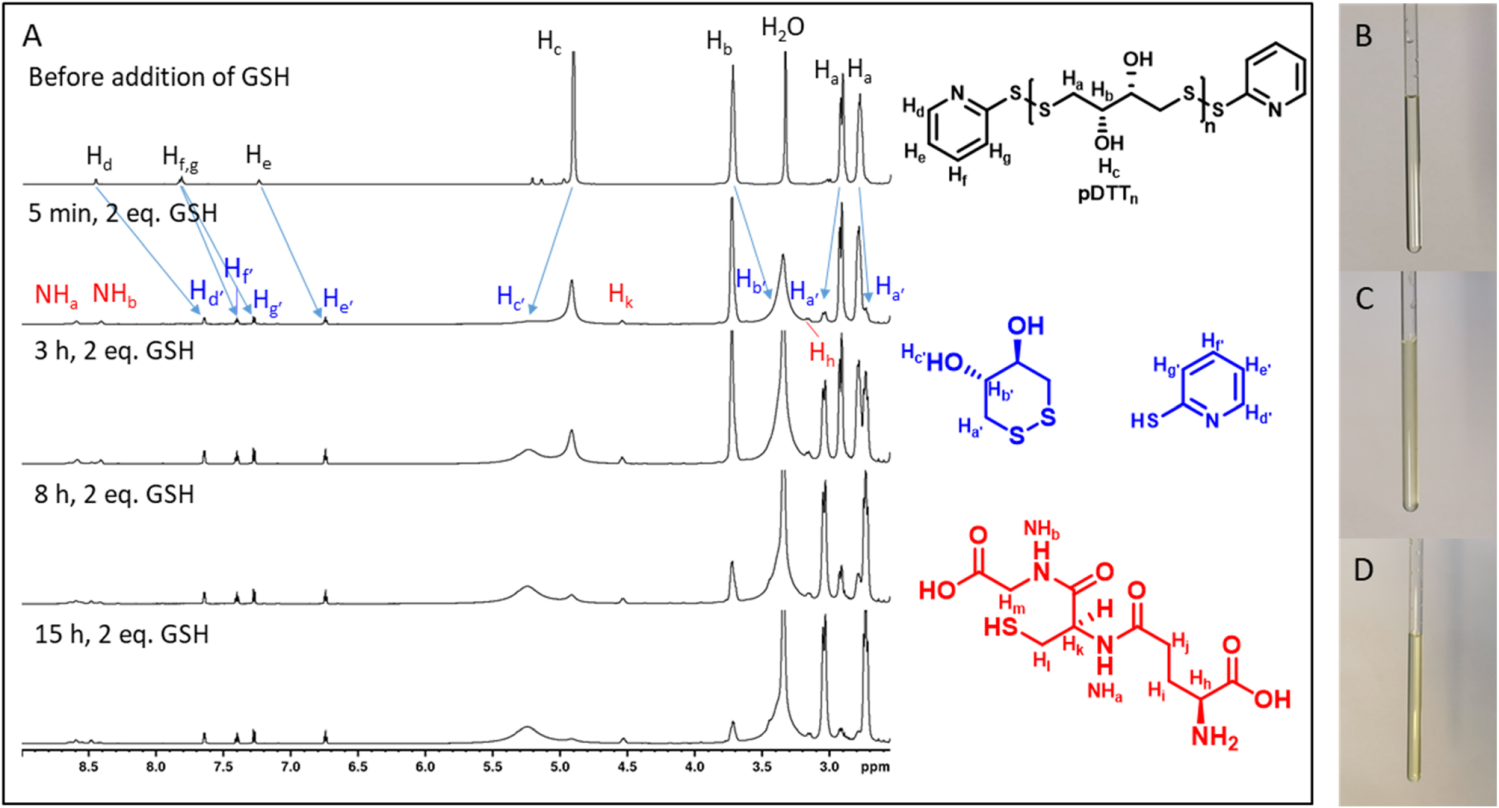
Degradation of pDTT_36_ with 2 eq. GSH (10.7 mM), ^1^H-NMR spectra before and after addition of GSH (5 min, 3 h, 8 h, 16 h) (A), clear solution of pDTT_36_ dissolved in d_6_-DMSO (B), turbid and light yellow solution of pDTT_36_ dissolved in d_6_-DMSO immediately after addition of GSH (C), clear and yellow solution of pDTT_36_ dissolved in d_6_-DMSO (D).

**Fig. 3:**
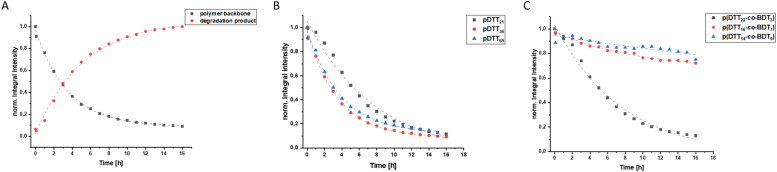
Normalized integral intensity of ^1^H-NMR of multiplett at 2.88 ppm (polymer backbone) and at 2.99 ppm (degradation product) (A), normalized integral intensity of ^1^H-NMR of multiplett at 2.88 ppm (polymer backbone) of pDTT_21_, pDTT_36_ and pDTT_69_ (B), normalized integral intensity of ^1^H-NMR of multiplett at 2.88 ppm (polymer backbone) of p(DTT_22_-*co*-BDT_3_), p(DTT_16_-*co*-BDT_7_) and p(DTT_14_-*co*-BDT_9_) (C).

To examine whether the polymers are suitable as basis for drug delivery systems their stability was tested at 37 °C at physiological concentrations of GSH. GPC experiments were conducted for all polymers at GSH concentrations of 2 μM and 10 mM in order to mimic the typical concentration in the bloodstream and the upper limit concentration within the cytoplasm.^39,40^ For pDTT_36_ a concentration of 2 mM GSH was tested as well which represents the lower limit of GSH found in healthy cells.^39^ The intensities of the polymer peaks were calculated in relation to the BHT signal, which is constant during the experiment (Fig. 1A, B and S13†). The polymer was observed to undergo slow degradation at a concentration of 2 μM GSH, as the relative intensity at the peak maximum decreased by 2% within 24 h after addition of GSH (Fig. 1A and C) and only by 14% within 72 h. This suggests a resistance not only to low concentrations of GSH, but also stability at 37 °C. Upon the particle's entry into the cell, there is a significant difference in the GSH concentration, which should lead to the immediate rupture of the particle and a complete degradation of the polymer. For this, the timeframe of the degradation should be as short as possible at elevated GSH levels. At a GSH concentration of 2 mM the polymer showed an increased degradation speed (35% in 24 h), however within the given time period the polymer did not fully degrade (Fig. 1C). The fastest degradation was detected at a GSH concentration of 10 mM (Fig. 1B and C). The intensity decreased by 87% within 24 h. It can be argued that due to the low solubility of GSH in organic solvents it is dispersed in DMAc, therefore the actual concentration of GSH might be lower and only the GSH in solution can contribute to the degradation of the polymer. The minor changes in the elution volume can be interpreted by understanding the self-immolative nature of the degradation mechanism: the chains do not partially degrade but in a chain reaction and this leads to a decrease in intensity rather than a decrease in hydrodynamic radius. As visualised in Fig. 1C the intensity decreases for all studied concentrations and as depicted before higher concentrations lead to a faster decay. All homo- and copolymers were treated with 10 mM GSH and all showed a degradation of at least 55% within 24 h (Fig. 1D). To further inspect the effect of GSH on the polymers NMR degradation studies were carried out. The polymers were dissolved in DMSO-d_6_ and analysed prior to the degradation. To reduce the effect of concentration changes, a pasteous mixture of GSH in DMSO-d_6_ (2 eq.) was added to the polymer solution (*e.g.* pDTT_36_: *c*_polymer_ = 5.44 mM, *c*_GSH_ = 10.7 mM). GSH was dissolved slowly during the reaction and NMR spectra were recorded immediately after addition of GSH. The CH_2_-backbone signal at 2.88 ppm was used to quantify the remaining polymer backbone, since this peak showed the least overlap (CH peak at 3.73 ppm overlaps with CH_2_ peak of GSH (Fig. 2A and S23†)). The cDTT degradation product was quantified using the signal at 2.99 ppm. Both integral values were analysed using the DMSO-d_6_ signal as an internal standard. In accordance with the DTT induced degradation shown by Pal *et al.*,^38^ the end-groups were cleaved first (Fig. 2A). This is also visible due to the yellow discoloration after the addition of GSH (Fig. 2C). DTT has two functional ends, which can immediately degrade the backbone by replacing the pyridylsulfide end-group. However, GSH, as a monothiol, must cleave off the end-group or attack in the middle of the polymer chain. The inhibited start of the degradation as well as the less well-resolved NMR spectrum immediately after addition can be explained by the poor solubility of GSH in DMSO-d_6_ (Fig. 2C). The observation of both backbone signals and the growing cDTT signal show an inverse trajectory, thereby delivering an internal validation of the degradation behaviour (Fig. 3A). In contrast to the GPC studies pDTT_36_ showed a more drastic degradation (Fig. 3B). All homopolymers showed a similar degradation rate and behaviour while the copolymers with higher BDT content p(DTT_14_-*co*-BDT_9_) and p(DTT_16_-*co*-BDT_7_) showed a degradation of only 20% (Fig. 3C). This behaviour is in good agreement with the findings of the Daasbjerg group who found pBDT to be less reactive towards DTT than pDTT without addition of bases.^35,38^ This is also reflected in their stability at *c*_GSH_ = 2 μM in the GPC experiments (Fig. S37†) and may be beneficial as this might render the particle more resistant in the bloodstream than their pDTT counterparts. McBride and Gillies found a similar degradation behaviour for their self-immolative linear polycarbamates, however they found a correlation between chain length and polymer half-life, that was not found in this study.^41^ However, unlike the polydisulfides studied here, the polycarbamates showed almost monodispersity. This has a strong influence on the accuracy of the half-life measurement and may be the reason for the missing correlation. Even though the GSH induced degradation is much slower than with DTT as a reducing agent, it still shows characteristics desirable for a burst release. Moreover, the polymers show only a slow degradation at *c*_GSH_ = 2 μM, which suggests an initial stability in the bloodstream. Since all polymers showed similar behaviour all are deemed suitable candidates for use in drug delivery systems. As the DTT-homopolymers showed the most efficient degradation, the following experiments were carried out using pDTT_36_ as a proof of concept.

### pDTT based nanoparticle preparation

The well-established solvent displacement method was used to prepare nanoparticles (NPs) composed of the polymer pDTT_36_. The hydrodynamic diameter of the resulting nanoparticles was approximately 180 ± 9 nm with a near-monodisperse size distribution reflected by a polydispersity index (PDI) around 0.10 ± 0.02 (Fig. S44†). Nanoparticles with a size of approximately 100 to 200 nm, as prepared in this study are commonly assumed to evade elimination by the liver and spleen, as well as being small enough to benefit from the enhanced permeability and retention (EPR) effect.^42^ Regarding the size of the prepared pDTT_36_-NPs they have the potential to provide a suitable carrier system for EPR-based targeted treatment of solid tumors. The zeta potential showed a slightly negative value close to zero (−4.3 ± 2.6 mV). Regarding electrostatic stabilisation of colloids it is assumed that an electrical potential of ±30 mV is required.^43^ Therefore, the zeta potential of the pDTT_36_-NPs was considerably too low to stabilize the colloidal system. Regardless, polymeric colloidal carriers can be stabilized also sterically by using polyvinyl alcohol (PVA).^44^ Thus, a stable pDTT_36_-nanocarrier system could be obtained. The resulting particles were visualized using scanning electron microscopy (SEM). The images showed spherical particles and confirmed a diameter of approximately 172 nm (see Fig. 5A, C, S49 and Table S2†).

### pDTT_36_-nanoparticle degradation

**Fig. 4:**
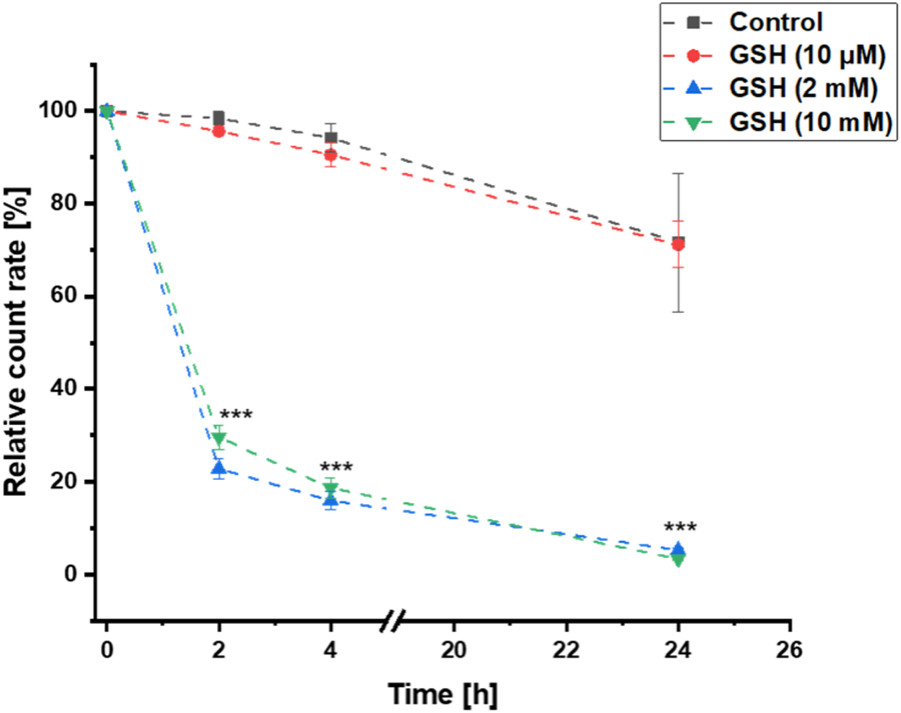
DLS count rate of PDTT_36_ particles during incubation with different GSH concentrations in relation to the initial value (mean ± SD; *n* = 3). Significant differences in relative count rate between intracellular and extracellular conditions were labelled (***p ≤ 0.001).

**Fig. 5:**
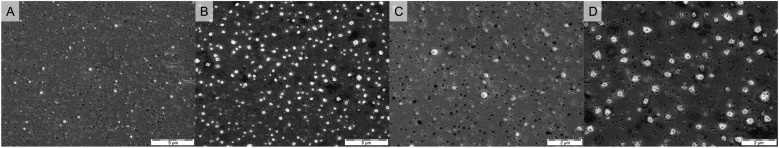
pDTT_36_ nanoparticles visualized by scanning electron microscopy at 5,000× magnification without treatment, scale bar = 5 μm (A) and after 24 h incubation with 10 mM glutathione, scale bar = 5 μm (B). pDTT_36_-nanoparticles visualized by scanning electron microscopy at 10 000× magnification without treatment scale bar = 2 μm (C) and after 24 h incubation with 10 mM glutathione, scale bar = 2 μm (D).

As previously described, the pure polymer pDTT_36_ could be degraded within 24 h by glutathione (GSH) concentrations between 2–10 mM, while remaining stable during incubation with 2 μM. To ensure that this property was not compromised by processing to nanoparticles, the decomposition of the colloidal pDTT_36_-carrier system was investigated. To test the particle stability within the scope of possible GSH concentrations in the extracellular matrix, a value at the upper reference range was selected.^40^ The pDTT_36_-NPs were incubated at 37 °C with various GSH concentrations to simulate extracellular (10 μM) and intracellular conditions (2–10 mM). In order to monitor the degradation of the NPs, the change in count rate during dynamic light scattering (DLS) measurement was considered.^45,46^ During incubation using high GSH concentrations the DLS count rate decreased to approximately 25% of the initial value within the first 2 h (Fig. 4). After 24 h the value reduced to 5%. Incubation in 10 μM GSH showed similar results compared to control samples without GSH with count rates ≥70% after 24 h. The size of the nanoparticles as well as their PDI remained stable without addition of GSH, whereas particles in a reducing environment (2–10 mM) rapidly swelled and became inhomogenous (Fig. S44†). This behaviour can be attributed to water intruding into the particles and lastly aggregation of the degradation products. To confirm the results of the DLS measurement, visualization was carried out using SEM (Fig. 5B and D). After incubation with 10 mM GSH, non-spheric fragments could be detected. However, these appeared to be melted and translucent. There were no more particles recognizable as before (Fig. 5A and C). Overall, the decomposition experiments indicated that the prepared NPs could be degraded solely with higher GSH concentrations and are therefore favorable decomposed for example in tumor tissue.

### Cell culture studies

**Fig. 6:**
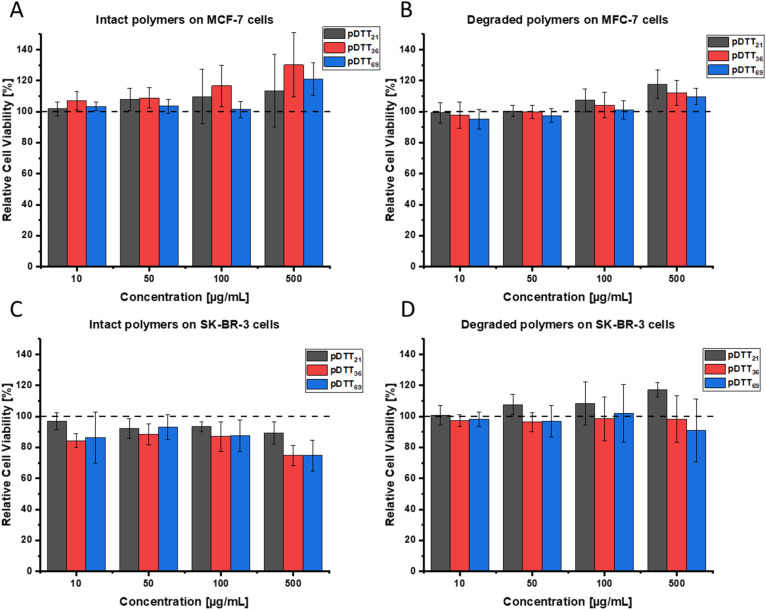
Comparison of the concentration dependent cytotoxic effects of intact polymers on MCF-7 cells (A), degraded polymers on MCF-7 cells (B), intact polymers on SK-BR-3 cells (C) and degraded polymers on SK-BR-3 cells (D). The relative cell viability was determined by WST-1 assay.

As the synthesized polymers are intended to be applied as drug delivery systems to breast cancer patients, it is necessary to prove that pDTT_21_, pDTT_36_, pDTT_69_ and their degradation products show no cytotoxic properties themselves, especially for surrounding tissues. Therefore, the WST-1 assay was conducted with two breast cancer cell lines (MCF-7 and SK-BR-3), that differ in expression of progesterone receptor, estrogen receptor and HER2. Neither the intact polymers nor their degradation products showed cytotoxicity in MCF-7 cells (Fig. 6A and C) or SK-BR-3 cells (Fig. 6B and D). Over the tested concentration range up to 500 μg mL^−1^ no detectable effects regarding cell viability were observed. These data underline the biosafety of the used polymers. Consequently, an effect of a future drug system can be solely attributed to an incorporated API and not to the pDTT polymer itself.

### Drug loading with nile red

**Fig. 7:**
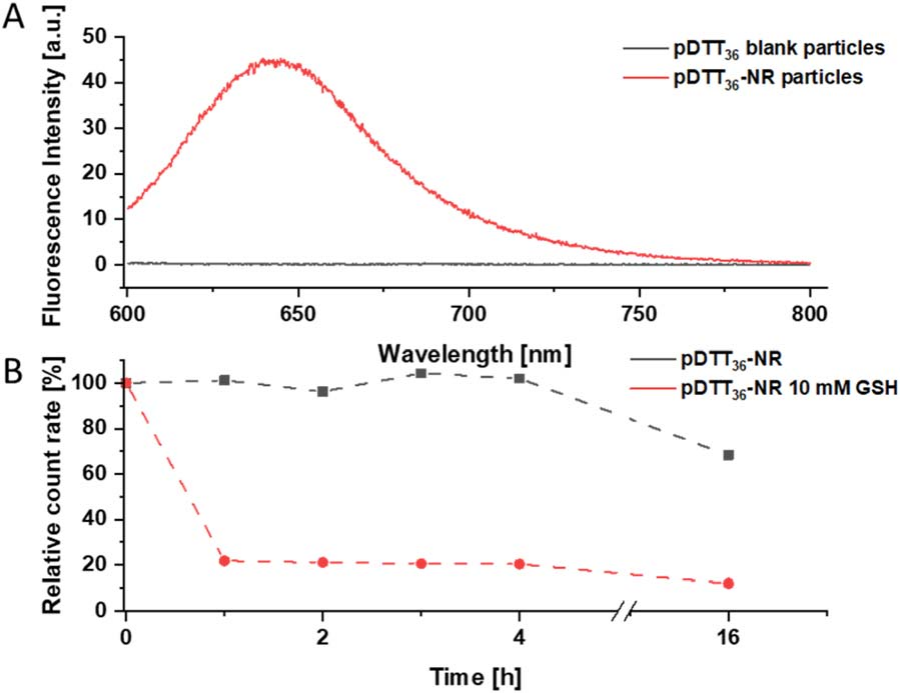
Fluorescence of unloaded (blank) pDTT_36_ particles and Nile red loaded particles (A); DLS count rate of pDTT_36_ particles during incubation with or without GSH in relation to the initial value (B).

Particles were loaded with the fluorescent dye Nile red that shows fluorescence emission at >600 nm in hydrophobic environments and fluorescence quenching in aqueous sytems.^47^ DLS analysis revealed similar diameters (182 nm) and PdI (<0.1) to the unloaded particles (Fig. S47†). A fluorescence signal at 640 nm was observed in the Nile red loaded particles, proving the successful loading of the dye (Fig. 7A). The degradation behaviour of the loaded particles did not differ significantly of that of the unloaded particles (Fig. 4 and 7B). A slight decrease in count rate was noted in TRIS buffer opposed to a pronounced decay in the presence of GSH. While the particles had maintained a uniform size and PDI in TRIS buffer, both increased rapidly when GSH was added (Fig. S47†). Immediately after each DLS measurement the fluorescence signal of the particles was measured. The particles showed good initial fluorescence and once treated with 10 mM GSH the fluorescence was quenched quickly (Fig. S45 and S46†). However, slow fluorescence quenching was observed in the untreated particles while the count rate did not suggest significant degradation. Since the particles show a slight swelling, and water intruding into the particles this can possibly be attributed to quenching the fluorescence. To confirm that fluorescence loss was not caused due to leakage of dye from the particle extensive stability tests, studies of drug loading capacity and release experiments will be conducted in follow-up publications with similar marker dyes, that do not suffer from fluorescence quenching (*e.g.* Lumogen Red®). Additionally, the loading of APIs and their cytotoxicity will be tested in the future.

## Conclusions

pDTT-homopolymers with different degrees of polymerization were successfully synthesized as well as p(DTT-*co*-BDT)-copolymers. It was shown that the pure polymers are degradable by glutathione. In two different breast cancer cell lines, no cytotoxicity was detectable by WST-1 assay for the homopolymers or their degradation products. To exclude mutagenicity, further experiments must be conducted. In addition, nanoparticles were successfully prepared from pDTT_36_ that are suitable for use in solid tumors in terms of size and size distribution. The nanoparticles were preferentially degraded by higher glutathione concentrations. Moreover, they were successfully loaded with the hydrophobic, fluorescent dye Nile red. Thus, the pDTT-nanocarrier could consequently be a useful “smart” drug delivery system to obtain a controlled drug release. However, it must be investigated whether active pharmaceutical ingredients can be embedded and selectively released under pathological conditions.

## Data availability

The data supporting this article have been included as part of the ESI.†

## Author contributions

Katharina Völlmecke: conceptualization, writing – original draft, methodology. Maurice Kramer: conceptualization, writing – original draft, methodology. Corinna Horky: methodology, writing. Oliver Dückmann: methodology. Dennis Mulac: methodology, review & editing. Klaus Langer: conceptualization, writing – review & editing, supervision, project administration. Dirk Kuckling: conceptualization, writing – review & editing, supervision, project administration.

## Conflicts of interest

There are no conflicts to declare.

## Supplementary Material

RA-014-D4RA07228F-s001
