# Supplementary material for: Self-immolative polydisulfides and their use as nanoparticles for drug delivery systems
Source: RSC Adv. 2024 Nov 7;14(48):35568–77. doi: 10.1039/d4ra07228f (PMC11541933; doi:10.1039/d4ra07228f)
Supplement: RA-014-D4RA07228F-s001 [file RA-014-D4RA07228F-s001.pdf]

Supplementary Information (SI) for RSC Advances.
This journal is © The Royal Society of Chemistry 2024

Supporting Information

Self-immolative Polydisulfides and their Use as Nanoparticles for Drug Delivery Systems

Katharina Völlmecke,[a] Maurice Kramer,[b] Corinna Horky,[b] Oliver Dückmann,[a] Dennis Mulac[b], Klaus Langer[b] and Dirk Kuckling *[a]

[a] Paderborn University, Warburger Straße 100, 33098 Paderborn, Germany
[b] Institute of Pharmaceutical Technology and Biopharmacy, University of Münster, Corrensstr. 48, D-48149 Münster, Germany

Contents

Materials & Methods 2
Materials 2
Synthesis of Homo- and Copolymers 2
Synthesis of $pDTT_{21}$ 2
Synthesis of $pDTT_{36}$ 2
Synthesis of $pDTT_{69}$ 3
Synthesis of $p(DTT_{14}$-co-$pBDT_9)$ 3
Synthesis of $p(DTT_{16}$-co-$pBDT_7)$ 3
Synthesis of $p(DTT_{22}$-co-$pBDT_3)$ 3
^{1}H-NMR Degradation Study 4
Polymer Analysis 6
GPC Analysis 6
NMR Analysis 7
DSC Analysis 11
Polymer Degradation 11
GPC results of $pDTT_{36}$ 11
GPC results of $pDTT_{69}$ 12
GPC results of $pDTT_{21}$ 13
NMR results of Homopolymer Degradation 14
Copolymer Degradation 17
GPC Results of $p(DTT_{14}$-*co*-$BDT_9)$ 17
GPC Results of $p(DTT_{16}$-*co*-$BDT_7)$ 18
GPC Results of $p(DTT_{22}$-*co*-$BDT_3)$ 19
^{1}H-NMR results of Copolymer Degradation 20
Particle Degradation 23
Blank Particles 23
Nile Red particles 23
SEM image analysis of blank particles 24

Materials & Methods

Materials

All materials were used without further purification. *N,N*-imethylacetamide (99.5%,), *N,N*-dimethylformamide (anhydrous, amine free, 99.9%), dithiothreitol (electrophoresis, 99%), and lithium bromide (anhydrous, 99+%) were purchased from Thermo Fisher. Dimethylsulfoxide-d_6 (99.8%) was obtained from Deutero GmbH. 2,2'-Dipyridyl disulfide (98%) was purchased from abcr. Glutathione (97%) was purchased from Alfa Aesar. 2,6-Di-tert-butyl-4-methylphenol (99%) was purchased from Fluka. Tetrahydrofuran (p.A.) was purchased from Stockmeier.

Synthesis of Homo- and Copolymers

Synthesis of $pDTT_{21}$

A schlenk flask was flushed with DPDS (2269 mg, 10.3 mmol, 1.03 eq.) and evacuated. The flask was then flushed with N_2 and DTT (1543 mg, 10.0 mmol, 1 eq.) was added. DMF was added to dissolve the solids (1.8 mL). After 1 h DMF (1.5 mL) was added to dilute the solution. The polymer was precipitated into four 30 mL centrifuge tubes with 1:1 chloroform/isohexane and centrifuged for 15 min. The precipitate was solved in DMF. The precipitation was repeated four times to give a light yellow solid that was dried in vacuo overnight. Yield: 859 mg; 53 %

$M_{n,NMR} \approx$ 3420 g mol^{-1}, $M_{n,GPC} \approx$ 4800 g mol^{-1}

^{1}H NMR (500 MHz, DMSO-d_6) δ ppm 2.78 (m, 43 H, CH_2), 2.92 (m, 43 H, CH_2), 3.72 (m, 46 H, CH), 4.91 (m, 43 H, OH), 7.24 (m, 2 H, CH), 7.82 (m, 4 H, CH), 8.46 (m, 2 H, CH)

^{13}C NMR (176 MHz, DMSO-d_6) δ ppm 41.9 (s, 1 C, CH_2), 42.0 (s, 1 C, CH_2), 70.5 (s, 1 C, CH), 70.6 (s, 1 C, CH), 119.3 (s, 1 C, CH), 121.1 (s, 1 C, CH), 137.8 (s, 1 C, CH), 149.5 (s, 1 C, CH), 159.4 (s, 1 C, CH)

Synthesis of $pDTT_{36}$

DTT (1853 mg, 12.01 mmol, 1 eq.) was placed in a N_2 flushed Schlenk flask, evacuated and flushed with N_2 again. DPDS (2673 mg, 12.13 mmol, 1.01 eq.) was added and the flask was evacuated and flushed with N_2 again. The solids were stirred and the powder turned from white to yellow. Anhydrous DMF (2.7 mL) was added and stirred at r.t. After 17 h the solution was turbid, DMF (1.5 mL) was added to resolve the precipitate. After 2 min the yellow solution was precipitated into four 30 mL centrifuge tubes with 1:1 chloroform/isohexane mixture and centrifuged for 15 min. The supernatant was removed and a sample of the solid was taken for NMR analysis. The precipitate was dissolved in DMF. The precipitation was repeated three times to give a light yellow solid that was dried in vacuo overnight. Yield: 1058.8 mg, 56%;

$M_{n,NMR} \approx$ 5700 g mol^{-1}, $M_{n,GPC} \approx$ 5600 g mol^{-1}

^{1}H NMR (500 MHz, DMSO-d_6) δ ppm 2.79 (m, 72 H, CH_2), 2.92 (m, 71 H, CH_2), 3.72 (br s, 74 H, CH), 4.91 (m, 73 H, OH), 7.24 (m, 2 H, CH), 7.83 (s, 4 H, CH), 8.46 (m, 2 H, CH)

^{13}C NMR (176 MHz, DMSO-d_6) δ ppm 41.9 (s, 1 C, CH_2), 42.0 (s, 1 C, CH_2), 70.5 (s, 1 C, CH), 70.6 (s, 1 C, CH), 119.3 (s, 1 C, CH), 121.1 (s, 1 C, CH), 137.8 (s, 1 C, CH), 149.5 (s, 1 C, CH), 159.4 (s, 1 C, CH)

Synthesis of $pDTT_{69}$

DPDS (2673 mg, 12.13 mmol, 1.01 eq.) was placed in a N_2 flushed Schlenk flask, evacuated and flushed with N_2. DTT (1854 mg, 12.02 mmol, 1 eq.) was added and the flask was evacuated and flushed with N_2 again. The solids were stirred and the powder turned from white to yellow. The mixture was cooled in an ice bath. Anhydrous DMF (2.7 mL) was added and stirred at 0 °C. After one hour the solution became turbid. After 24 h the suspension was diluted with DMF (0.5 mL) and precipitated into four 30 mL centrifuge tubes with 1:1 chloroform/isohexane mixture and centrifuged for 15 min. The precipitate was dissolved in DMF and precipitated again. The precipitation was repeated four times to give a light yellow solid that was dried in vacuo overnight. Yield: 1075.5 mg, 58%; $M_{n,NMR} \approx$ 10730 g mol^{-1}, $M_{n,GPC} \approx$ 7300 g mol^{-1}

^1H NMR (500 MHz, DMSO-d_6) δ ppm 2.78 (m, 138 H, CH_2), 2.92 (m, 137 H, CH_2), 3.72 (br s, 145 H, CH), 4.91 (br s, 136 H, OH), 7.24 (m, 2 H, CH), 7.82 (m, 4 H, CH), 8.45 (m, 2 H, CH)

^{13}C NMR (176 MHz, DMSO-d_6) δ ppm 41.9 (s, 1 C, CH_2), 42.0 (s, 1 C, CH_2), 70.5 (s, 1 C, CH), 70.6 (s, 1 C, CH), 119.3 (s, 1 C, CH), 121.1 (s, 1 C, CH), 137.8 (s, 1 C, CH), 149.5 (s, 1 C, CH), 159.4 (s, 1 C, CH)

Synthesis of $p(DTT_{14}$-co-$pBDT_9)$

BDT (1034 mg, 8.45 mmol, 1 eq.) was placed in a N_2 flushed Schlenk flask. DTT (1854 mg, 12.02 mmol, 1 eq.) was added. Anhydrous DMF (4 mL) was added and DPDS (4777 mg, 21.68 mmol, 2.57 eq) was added as soon as DTT was completely dissolved. Then anhydrous DMF (2 mL) was added. The mixture was cooled by an ice bath for 5 min. After 20 h the mixture was diluted with DMF (1.5 mL) and precipitated into four 30 mL centrifuge tubes with 1:1 chloroform/isohexane mixture and centrifuged for 15 min. The precipitate was dissolved in a DMF-THF mixture and precipitated again. The precipitation was repeated four times to give a yellow solid that was dried in vacuo overnight. Yield: 687.8 mg, 21%; $M_{n,NMR} \approx$ 3430 g mol^{-1}, $M_{n,GPC} \approx$ 3600 g mol^{-1}

^1H NMR (500 MHz, DMSO-d_6) δ ppm 1.73 (m, 37 H), 2.76 (m, 57 H), 2.91 (m, 36 H), 3.73 (m, 29 H), 4.91 (m, 28 H), 7.24 (m, 2 H), 7.82 (m, 4 H), 8.45 (m, 2 H)

Synthesis of $p(DTT_{16}$-co-$pBDT_7)$

BDT (721 mg, 5.89 mmol, 1 eq.) was placed in a N_2 flushed Schlenk flask. DTT (2122 mg, 13.76 mmol, 2.33 eq.) was added. Anhydrous DMF (4 mL) was added and DPDS (4436 mg, 20.13 mmol, 3.42 eq) was added as soon as DTT was completely dissolved. Anhydrous DMF (2 mL) was added. The mixture was cooled by an ice bath for 5 min. After 17 h the mixture was diluted with DMF (1.5 mL) and precipitated into four 30 mL centrifuge tubes with 1:1 chloroform/isohexane mixture and centrifuged for 15 min. The precipitate was dissolved in a DMF-THF mixture and precipitated again. The precipitation was repeated four times to give a yellow solid that was dried in vacuo overnight. Yield: 990.3 mg, 33%; $M_{n,NMR} \approx$ 3500 g mol^{-1}, $M_{n,GPC} \approx$ 3900 g mol^{-1}

^1H NMR (500 MHz, DMSO-d_6) δ ppm 1.74 (m, 27 H), 2.76 (m, 57 H), 2.91 (m, 36 H), 3.74 (m, 32 H), 4.92 (m, 31 H), 7.25 (m, 2 H), 7.83 (m, 4 H), 8.46 (m, 2 H)

Synthesis of $p(DTT_{22}$-co-$pBDT_3)$

BDT (381 mg, 3.12 mmol, 1 eq.) was placed in a N_2 flushed Schlenk flask. DTT (2673 mg, 17.33 mmol, 5.56 eq.) was added. Anhydrous DMF (4 mL) was added and DPDS (4617 mg, 20.96 mmol, 6.72 eq) was added as soon as DTT was completely dissolved. Then anhydrous DMF (2 mL) was added. The mixture was cooled by an ice bath for 5 min. After 4 h the mixture was diluted with DMF (1.5 mL) and precipitated into four 30 mL centrifuge tubes with 1:1 chloroform/isohexane mixture and centrifuged for 15 min. The precipitate was dissolved in a DMF-THF mixture and precipitated again. The

precipitation was repeated four times to give a yellow solid that was dried in vacuo overnight. Yield: 1282.8 mg, 40%; $M_{n,NMR}$≈ 3930 g mol^{-1}, $M_{n,GPC}$≈ 3800 g mol^{-1}

^{1}H NMR (500 MHz, DMSO-d_6) δ ppm 1.74 (m, 13 H), 2.78 (br d, J=9.4 Hz, 50 H), 2.92 (br d, J=2.1 Hz, 38 H), 3.73 (br s, 44 H), 4.92 (m, 44 H), 7.25 (m, 2 H), 7.83 (m, 4 H), 8.46 (m, 2 H)

^{1}H-NMR Degradation Study

Approximately 20 mg of polymer were dissolved in 600 µl DMSO-d_6 (precise concentrations of polymer concentrations in Table S1). ^{1}H-NMR spectrum was recorded. A GSH stock solution was prepared ($c_{GSH,stock}$ see Table S1) and 2 equivalents in respect to the polymer were added (volumes see Table S1) an the tube was shaken to mix. ^{1}H-NMR spectrum was recorded immediately after addition at 30 °C internal temperature.

Table S1: Concentrations and volumes used for ^{1}H-NMR degradation experiment, GSH stock solution was added to polymer solution to achieve c_{GSH} in solution

$pDTT_{21}$	$c_{Polymer}$ [mmol L^{-1}]	8.78
	$c_{GSH,stock}$ [mmol L^{-1}]	741.87
	c_{GSH} [mmol L^{-1}]	17.15
	volume GSH stock added [µL]	14.2
$pDTT_{36}$	$c_{Polymer}$ [mmol L^{-1}]	5.44
	$c_{GSH,stock}$ [mmol L^{-1}]	670.29
	c_{GSH} [mmol L^{-1}]	10.70
	volume GSH stock added [µL]	9.7
$pDTT_{69}$	$c_{Polymer}$ [mmol L^{-1}]	3.56
	$c_{GSH,stock}$ [mmol L^{-1}]	533.63
	c_{GSH} [mmol L^{-1}]	7.02
	volume GSH stock added [µL]	8.0
$p(DTT_{22}$-co-$BDT_3)$	$c_{Polymer}$ [mmol L^{-1}]	8.04
	$c_{GSH,stock}$ [mmol L^{-1}]	709.34
	c_{GSH} [mmol L^{-1}]	15.71
	volume GSH stock added [µL]	13.6
$p(DTT_{16}$-co-$BDT_7)$	$c_{Polymer}$ [mmol L^{-1}]	8.98
	$c_{GSH,stock}$ [mmol L^{-1}]	676.80
	c_{GSH} [mmol L^{-1}]	17.49
	volume GSH stock added [µL]	15.9
$p(DTT_{14}$-co-$BDT_9)$	$c_{Polymer}$ [mmol L^{-1}]	8.10

$c_{GSH,stock}$ [mmol L^{-1}]	683.30
c_{GSH} [mmol L^{-1}]	15.82
volume GSH stock added [µL]	14.2

Polymer Analysis

GPC Analysis

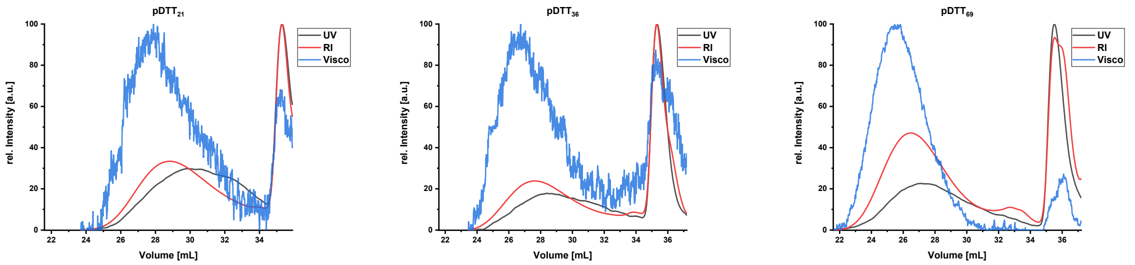

Figure S1: Elution curves of homopolymer pDTT$_{21}$, pDTT$_{36}$, and pDTT$_{69}$ in DMAc + 0.5 g L^{-1} LiBr, BHT internal standard signal at 35.2 mL

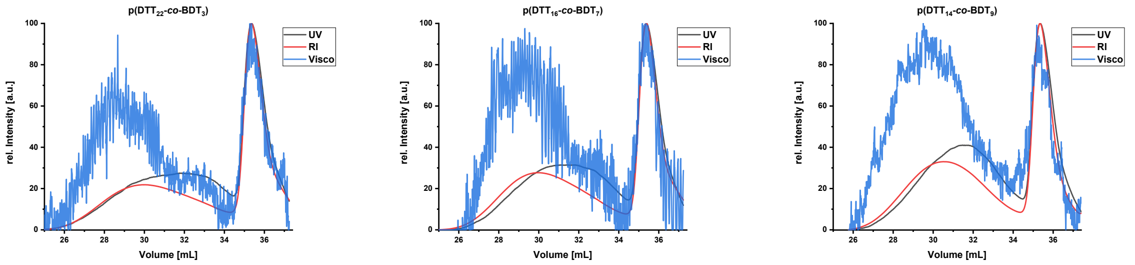

Figure S2: Elution curves of copolymer ^{1}H-NMR of p(DTT$_{22}$-co-BDT$_{3}$), ^{1}H-NMR of p(DTT$_{16}$-co-BDT$_{7}$), and ^{1}H-NMR of p(DTT$_{14}$-co-BDT$_{9}$) in DMAc + 0.5 g L^{-1} LiBr, BHT internal standard signal at 35.2 mL

NMR Analysis

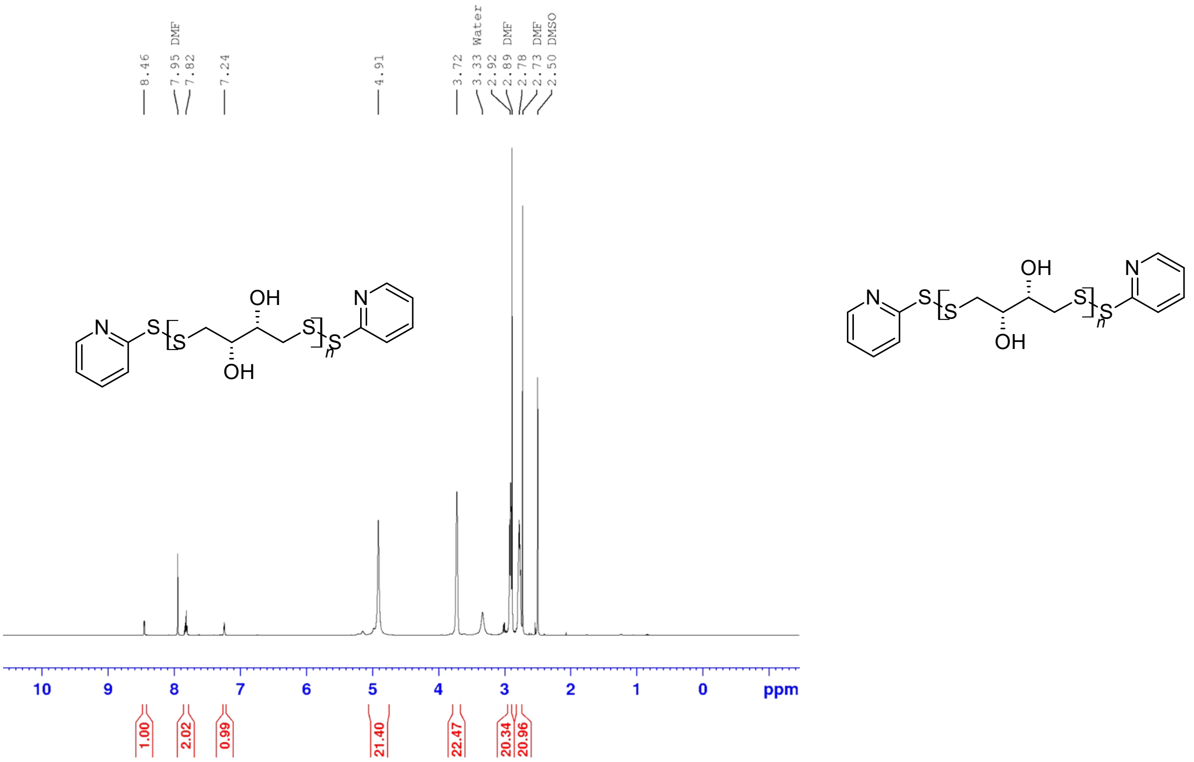

Figure S3: ^{1}H-NMR of pDTT$_{21}$

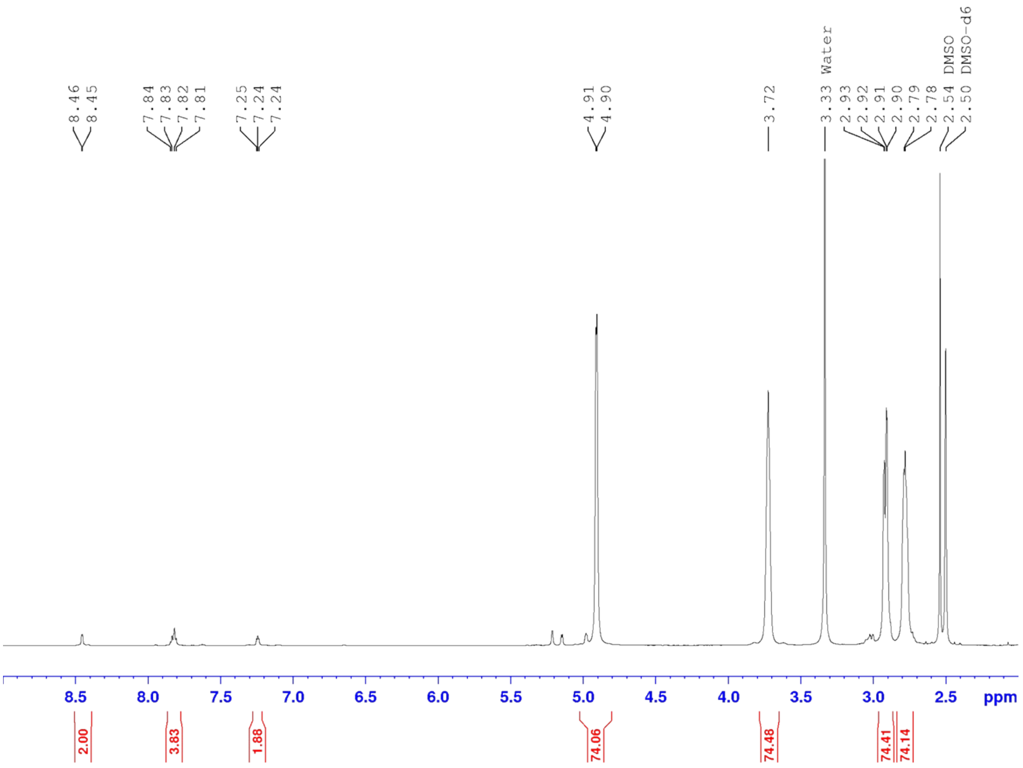

Figure S4: ^{1}H-NMR of pDTT$_{36}$ (purified by dialysis)

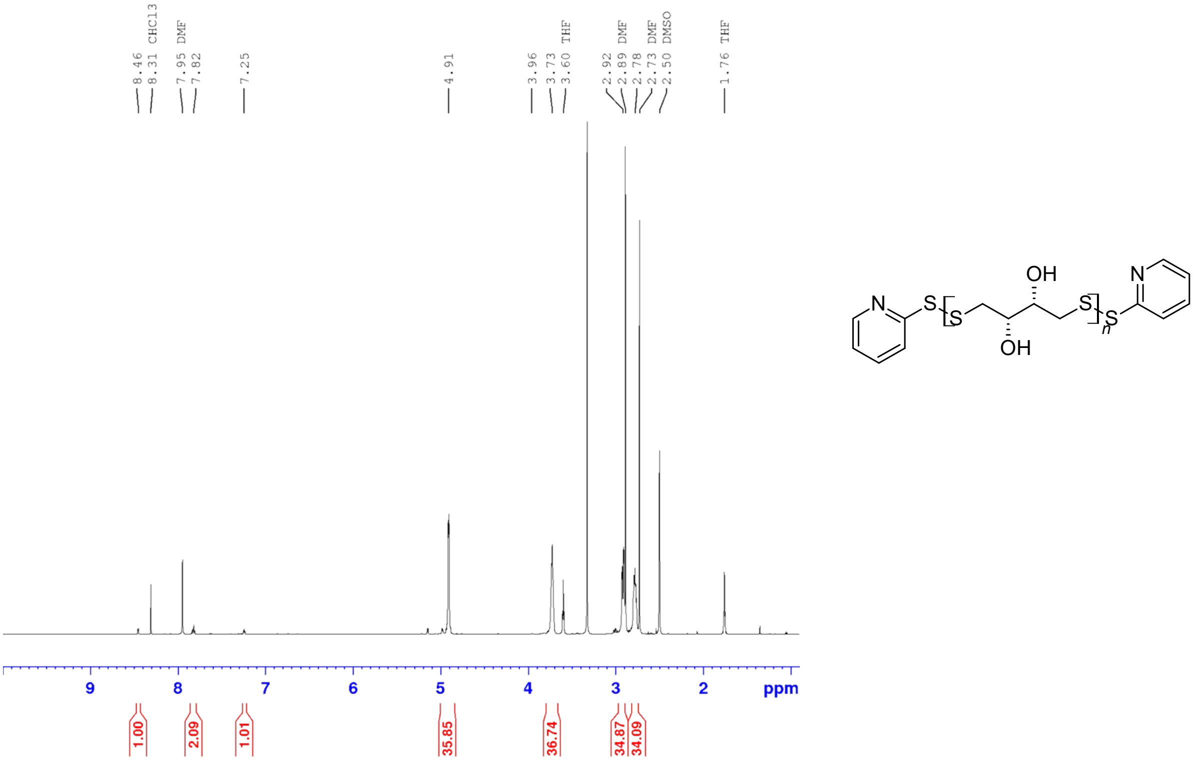

Figure S5: ^{1}H-NMR of pDTT$_{36}$

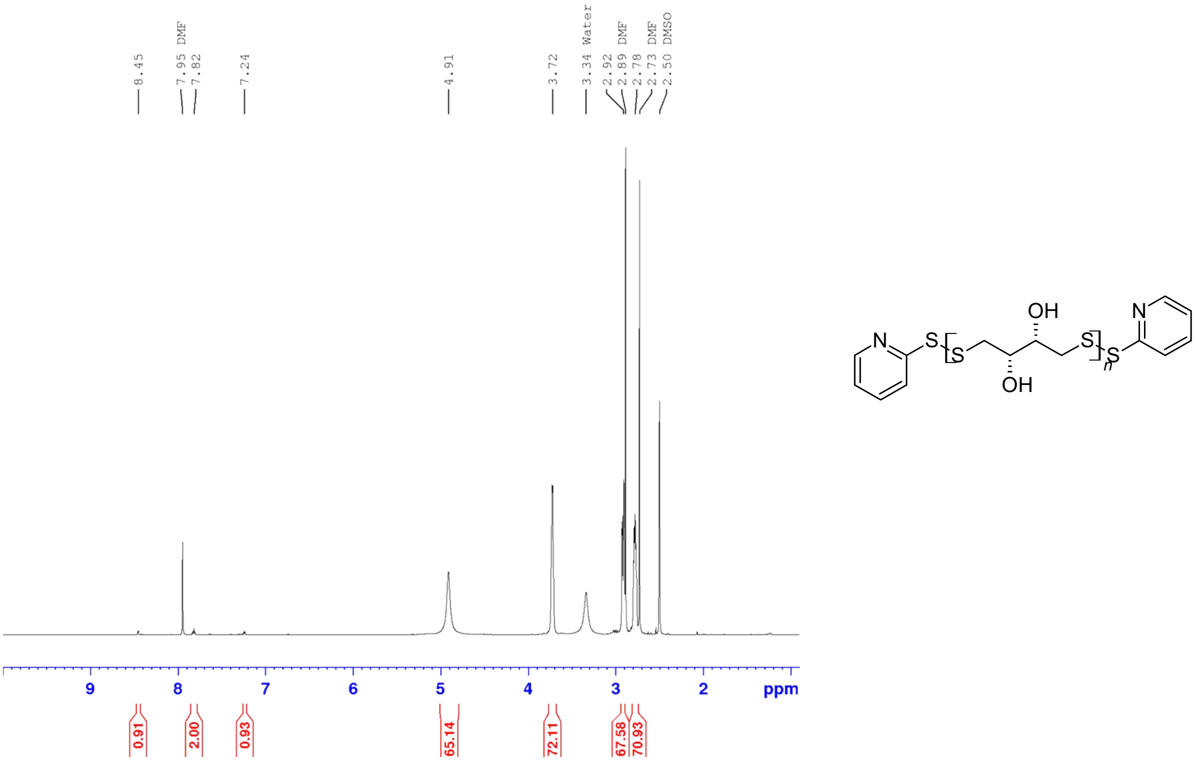

Figure S6: ^{1}H-NMR of pDTT$_{69}$

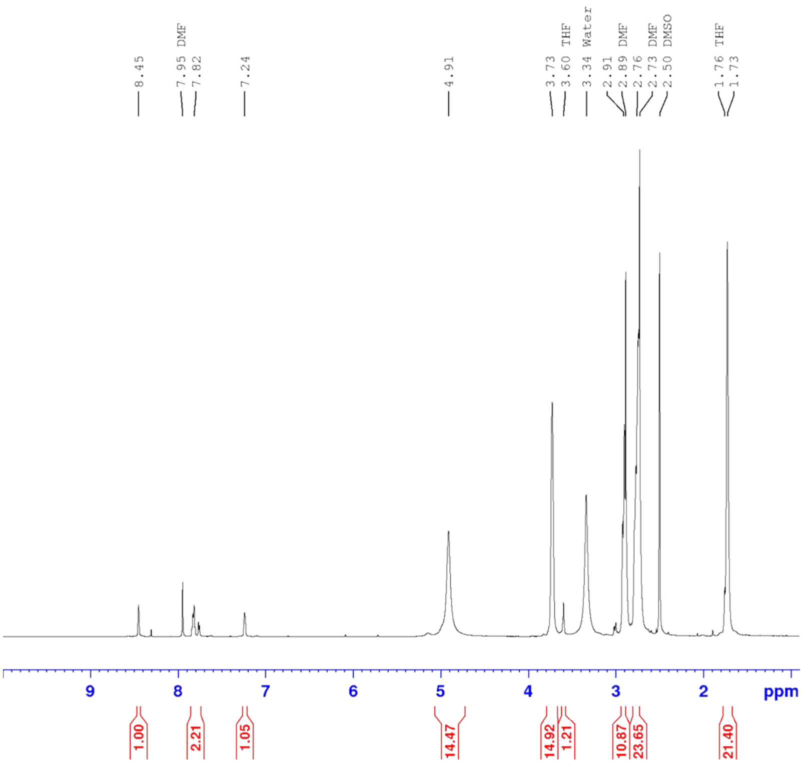

Figure S7: ¹H-NMR of p(DTT$_{14}$-co-BDT$_{9}$)

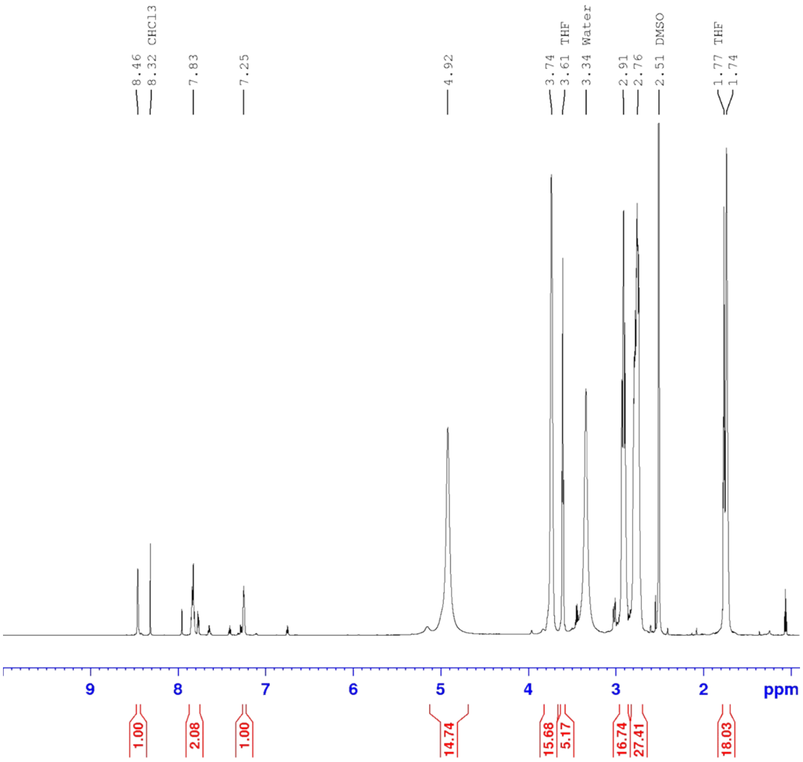

Figure S8: ¹H-NMR of p(DTT$_{16}$-co-BDT$_{7}$)

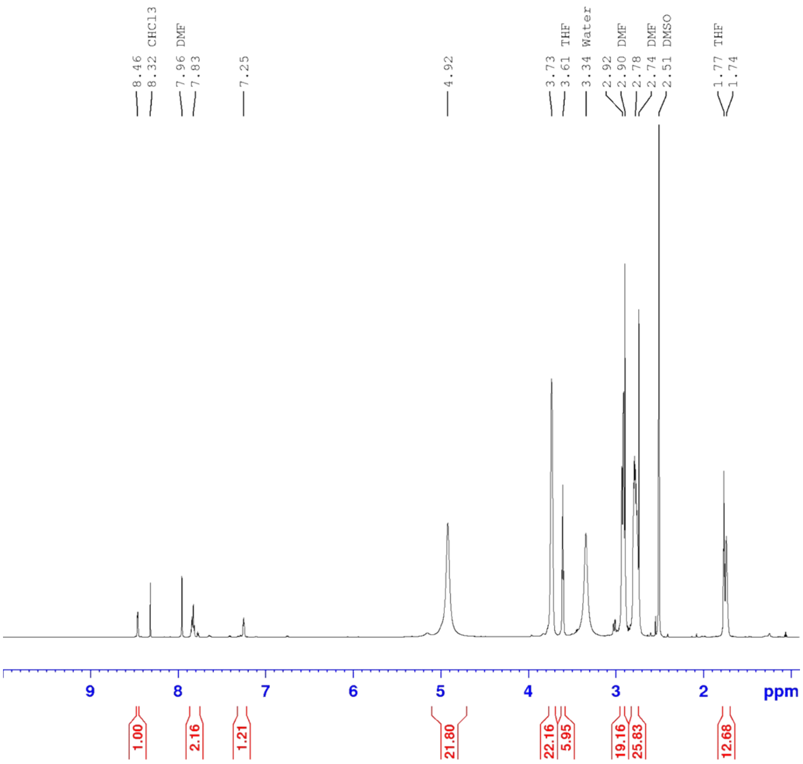

Figure S9: ^{1}H-NMR of p(DTT$_{22}$-co-BDT$_{3}$)

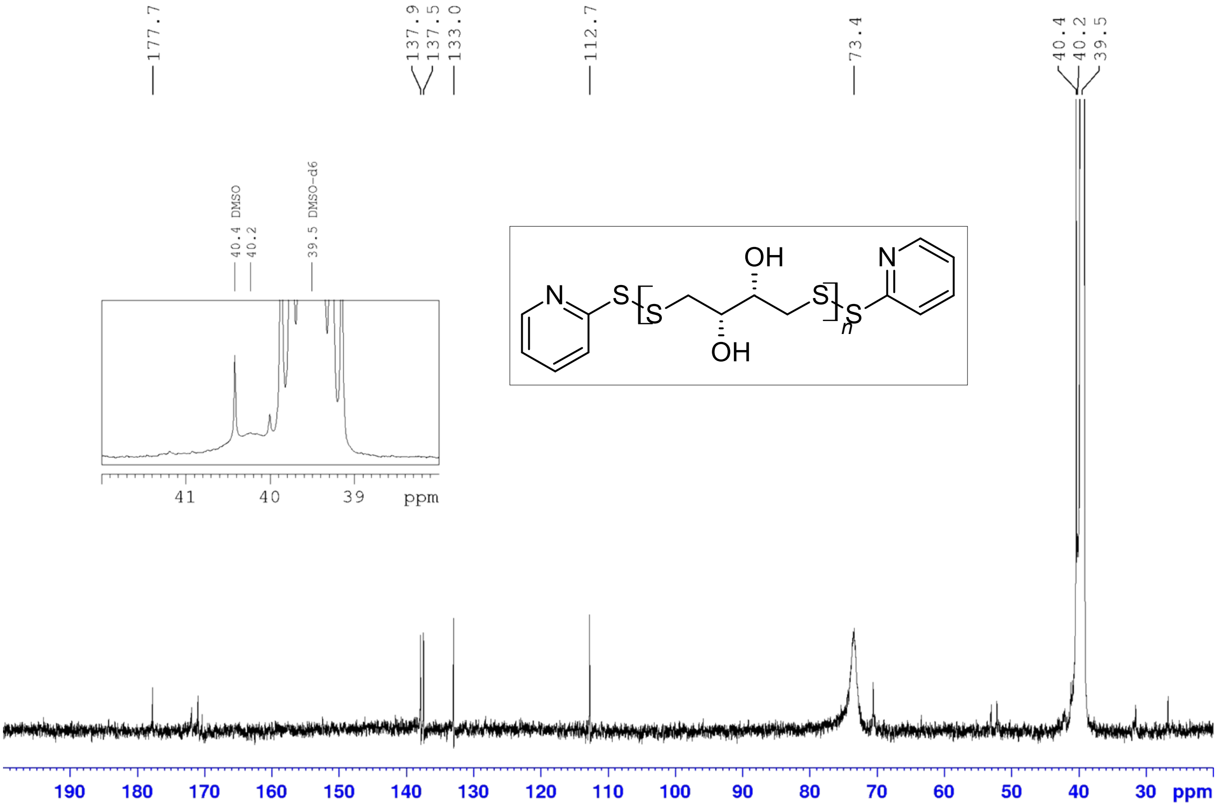

Figure S10: ^{13}C-NMR of pDTT$_{36}$

DSC Analysis

DSC analyses of the polymer samples were conducted using Netzsch DSC 204 F1 Phönix at a heating rate of 10 K/min under nitrogen atmosphere.

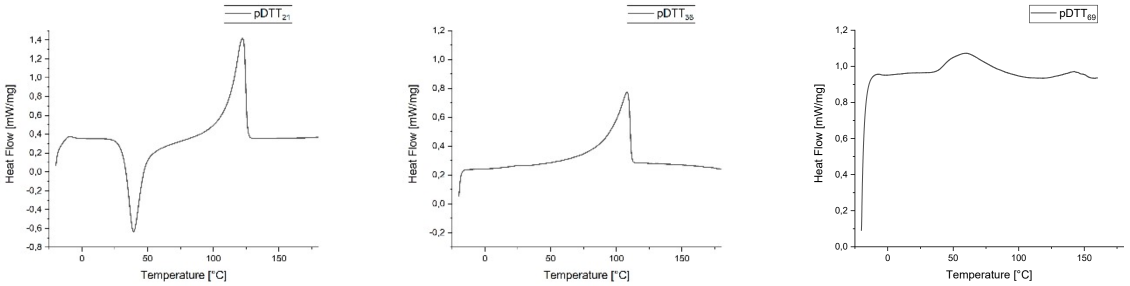

Figure S11: DSC Traces of homopolymers $pDTT_{21}$, $pDTT_{36}$ and $pDTT_{69}$

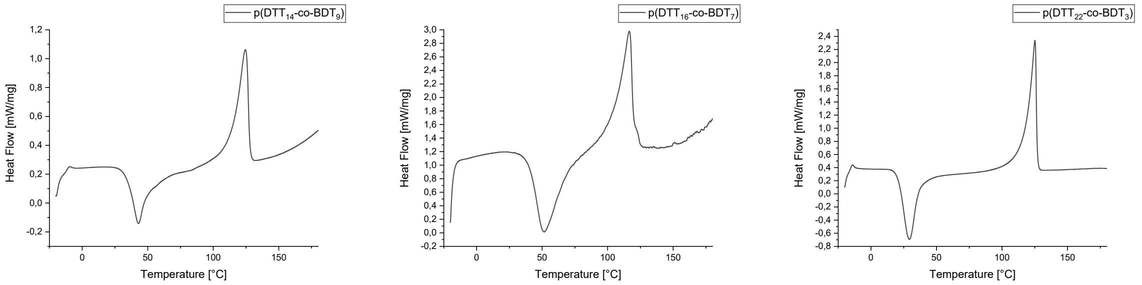

Figure S12: DSC Traces of copolymers $p(DTT_{14}$-co-$BDT_9)$, $p(DTT_{16}$-co-$BDT_7)$ and $p(DTT_{22}$-co-$BDT_3)$

Polymer Degradation

GPC results of $pDTT_{36}$

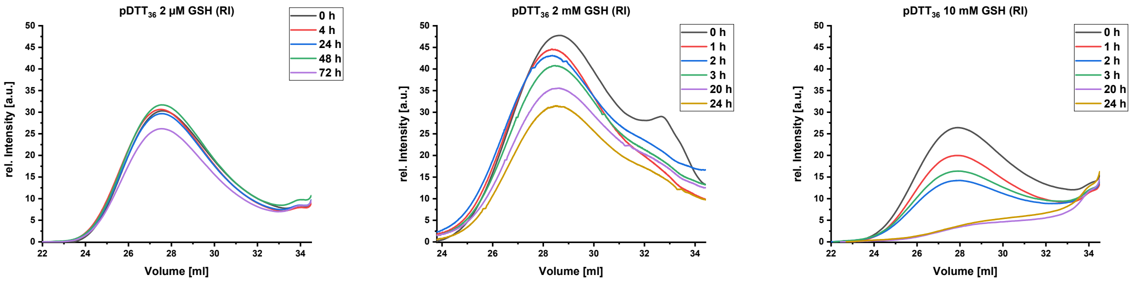

Figure S13: Degradation of $pDTT_{36}$ RI Detector

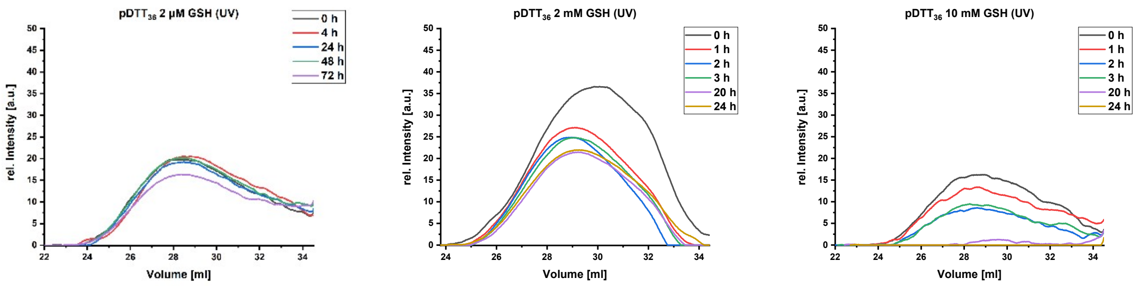

Figure S14: Degradation of $pDTT_{36}$ UV Detector

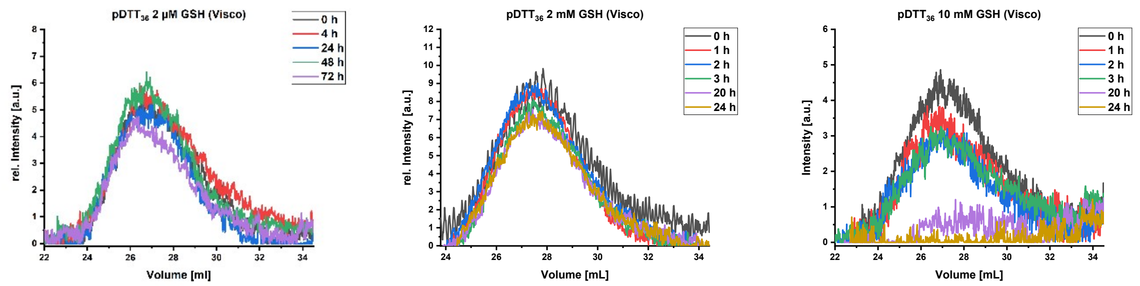

Figure S15: Degradation of $pDTT_{36}$ Viscosity Detector

GPC results of $pDTT_{69}$

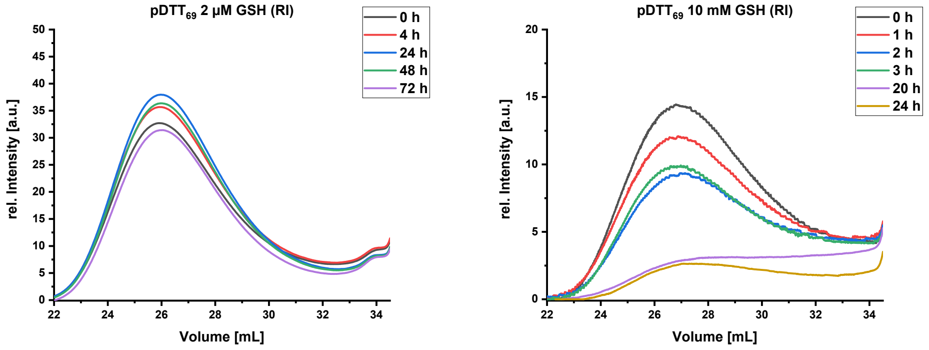

Figure S16: Degradation of $pDTT_{69}$RI Detector

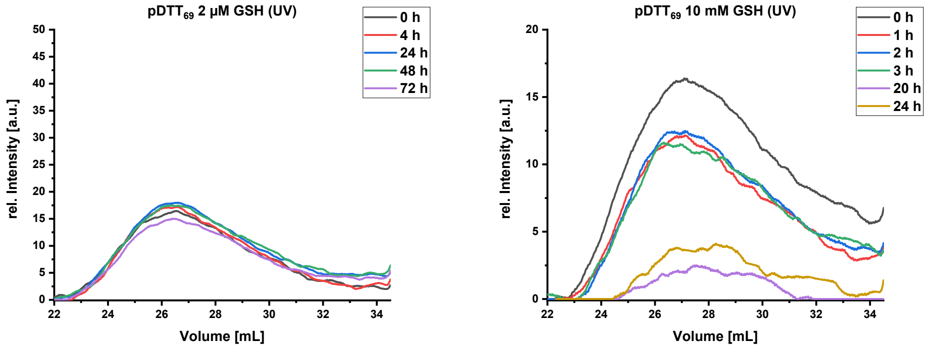

Figure S17: Degradation of $pDTT_{69}$ UV Detector

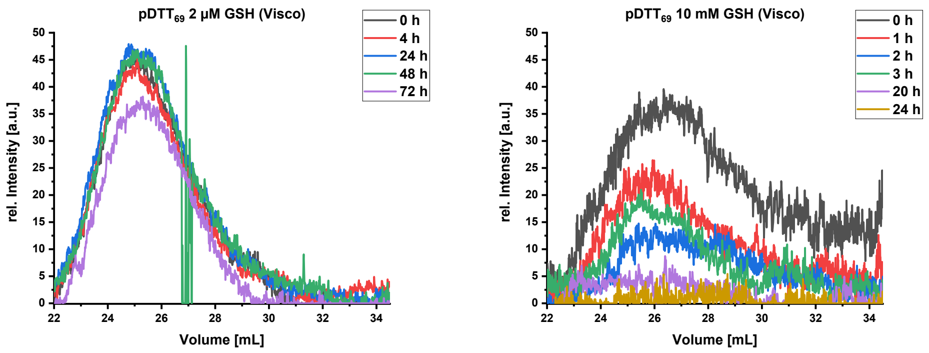

Figure S18: Degradation of $pDTT_{69}$ UV Detector

GPC results of $pDTT_{21}$

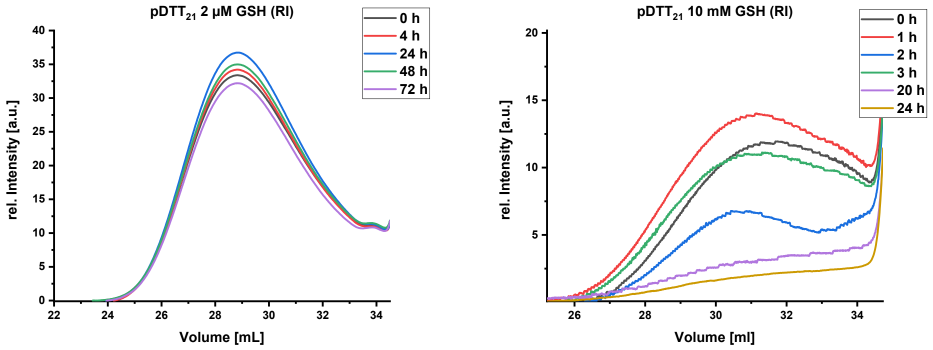

Figure S19: Degradation of $pDTT_{21}$ RI Detector

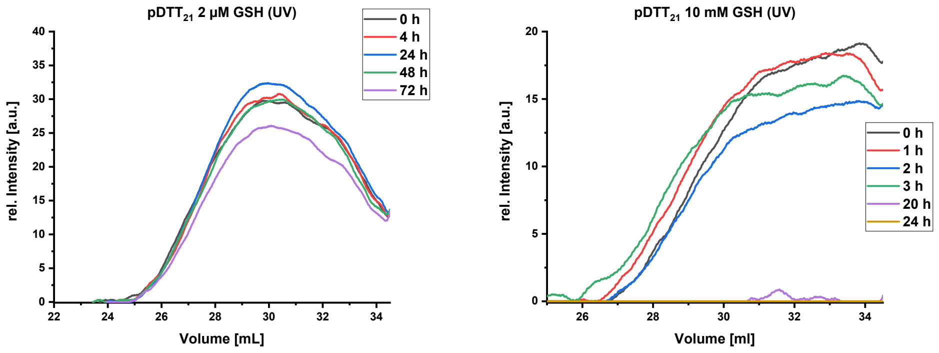

Figure S20: Degradation of $pDTT_{21}$ UV Detector

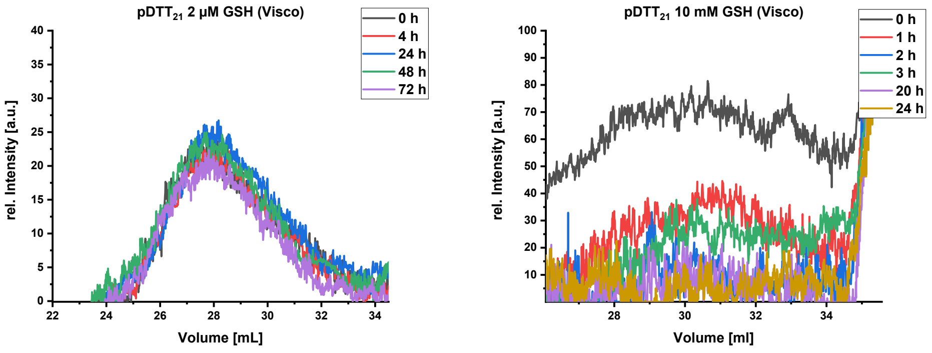

Figure S21: Degradation of $pDTT_{21}$ Visco Detector

NMR results of Homopolymer Degradation

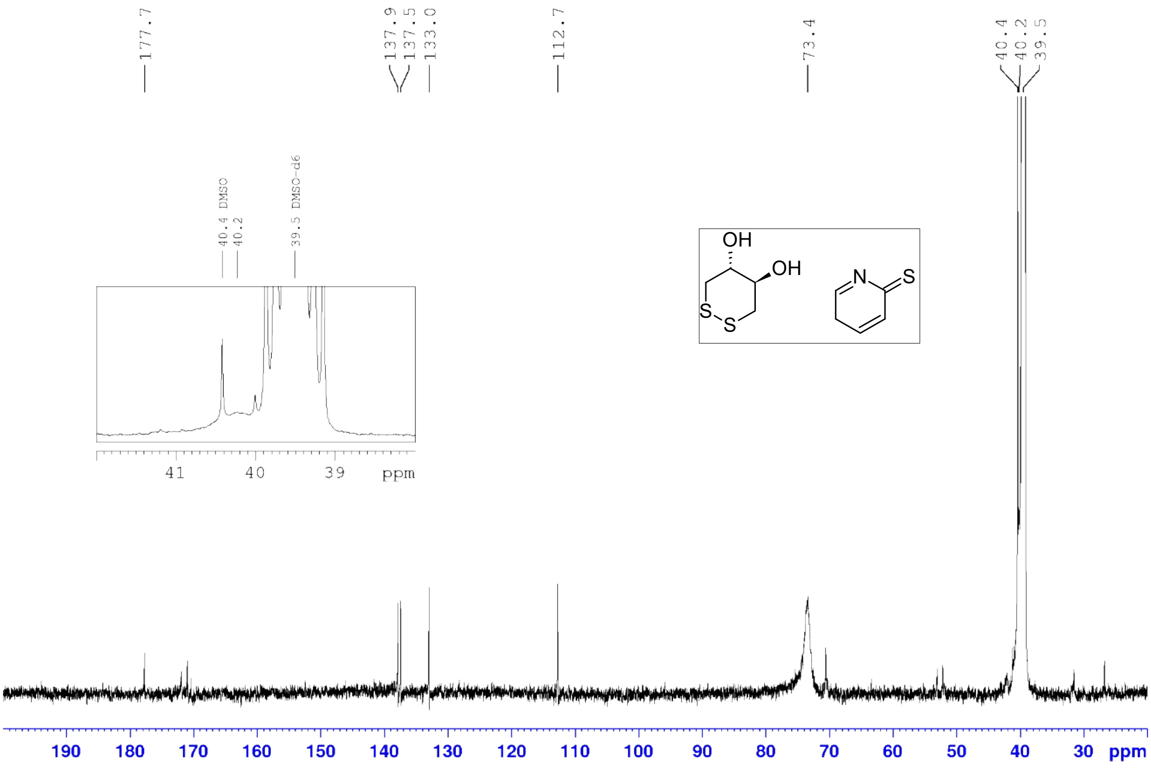

Figure S22: ^{13}C of degraded pDTT$_{36}$

^{13}C NMR (176 MHz, DMSO-d_6) δ ppm 40.2 (s, 1 C, CH_2), 112.75 (s, 1 C, CH), 133.0 (s, 1 C, CH), 137.5 (s, 1 C, CH), 137.9 (s, 1 C, CH), 177.7 (s, 1 C, C)

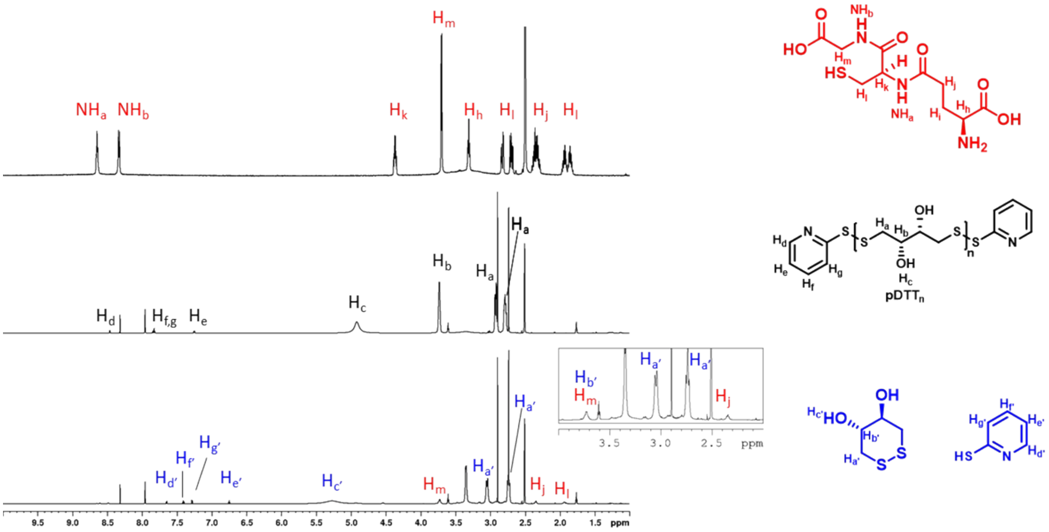

Figure S23: ^1H-NMR spectra of degradation of pDTT induced by GSH, Top spectrum: GSH, middle spectrum: pDTT, bottom spectrum: fully degraded pDTT with GSH

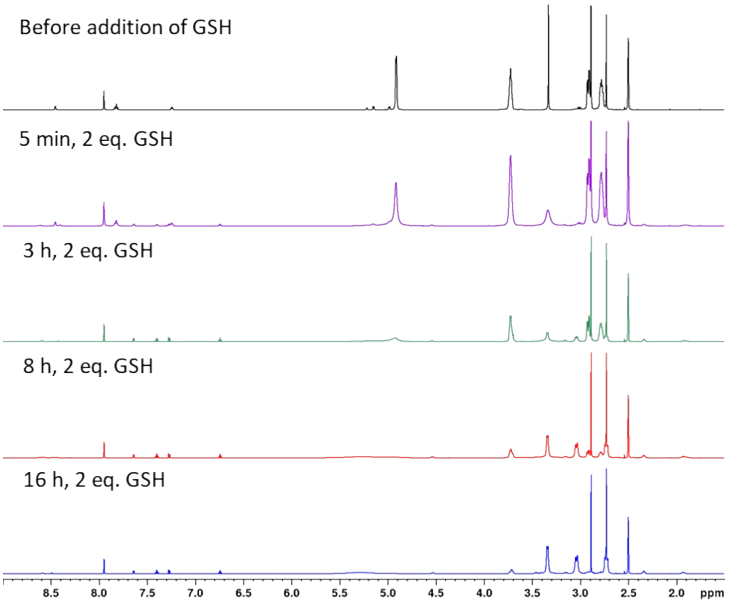

Figure S24: ¹H-NMR study of degradation of pDTT$_{21}$ by 2 eq. GSH, spectra recorded after 5 min, 3 h, 8 h and 16 h

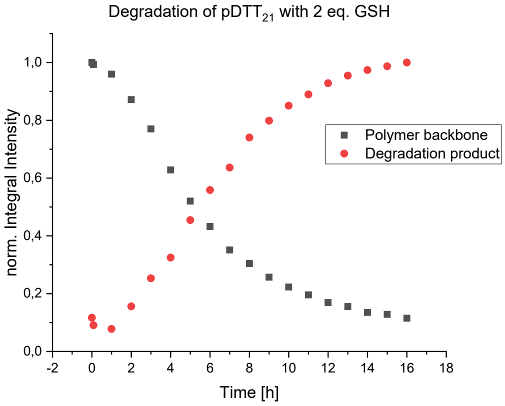

Figure S25: ¹H-NMR study of degradation of pDTT$_{69}$ by 2 eq. GSH. Integral Intensities normalized to maximum value before addition of GSH (backbone) and after 16 h (degradation product), spectra recorded 5 min after addition, then hourly

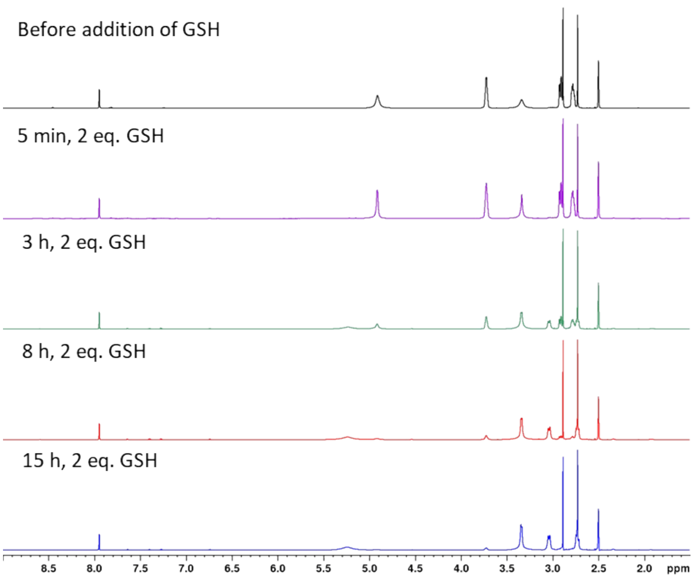

Figure S26: ^{1}H-NMR study of degradation of pDTT$_{69}$ by 2 eq. GSH, spectra recorded after 5 min, 3 h, 8 h and 15 h

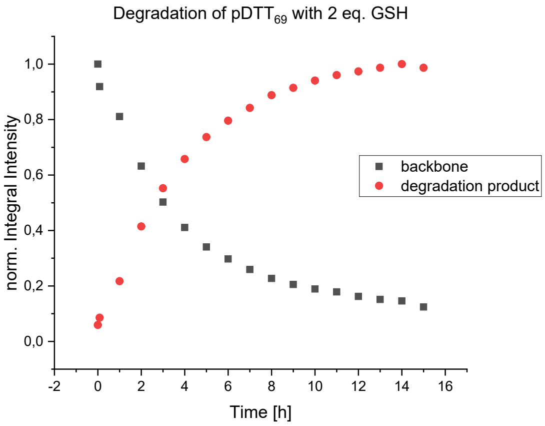

Figure S27: ^{1}H-NMR study of degradation of pDTT$_{69}$ by 2 eq. GSH. Integral Intensities normalized to maximum value before addition of GSH (backbone) and after 15 h (degradation product), spectra recorded 5 min after addition, then hourly

Copolymer Degradation

GPC Results of p(DTT_{14}-*co*-BDT_9)

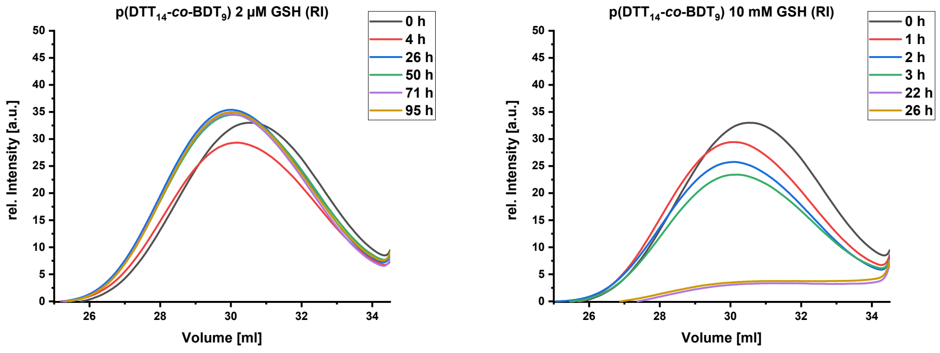

Figure S28: Degradation of p(DTT_{14}-co-BDT_9) RI Detector

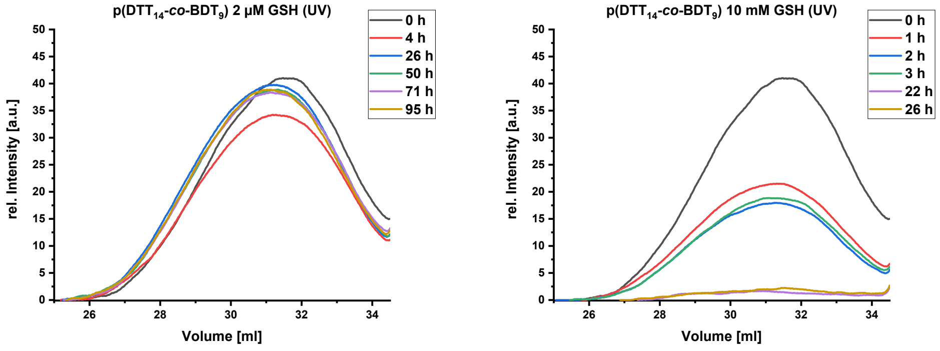

Figure S29: Degradation of p(DTT_{14}-co-BDT_9) UV Detector

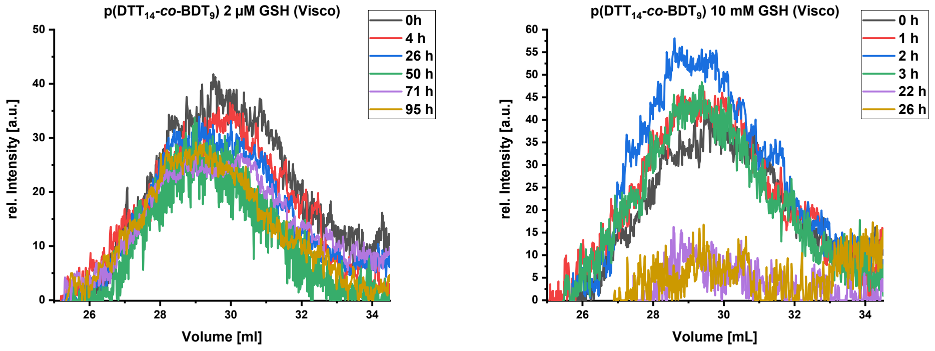

Figure S30: Degradation of p(DTT_{14}-co-BDT_9) Visco Detector

GPC Results of p(DTT_{16}-*co*-BDT_7)

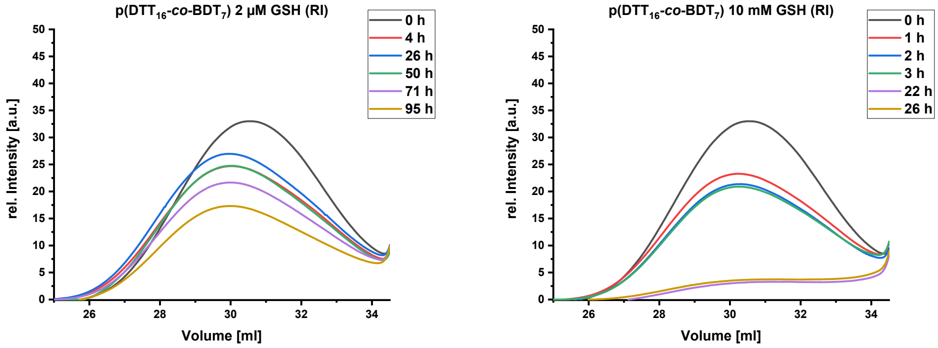

Figure S31:Degradation of p(DTT_{16}-co-BDT_7) RI Detector

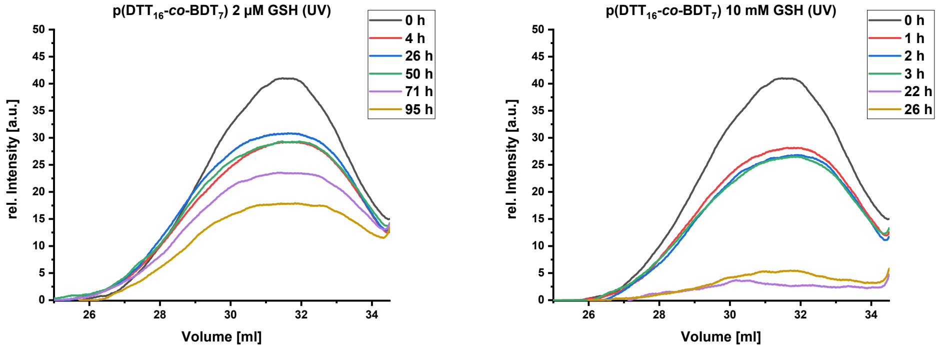

Figure S32: Degradation of p(DTT_{16}-co-BDT_7) UV Detector

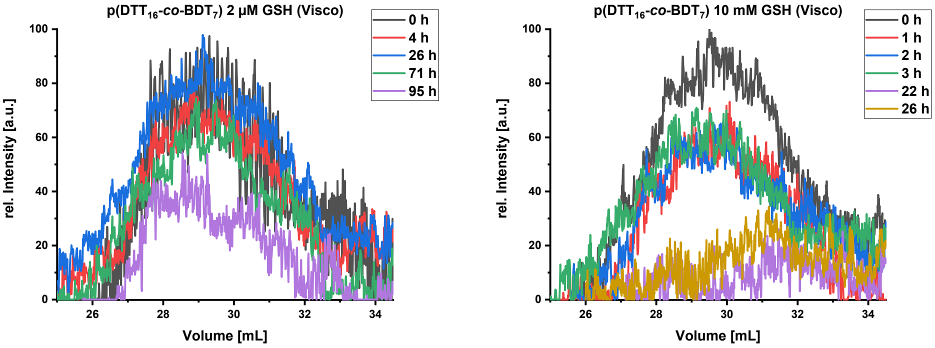

Figure S33: Degradation of p(DTT_{16}-co-BDT_7) Viscosity Detector

GPC Results of p(DTT_{22}-*co*-BDT_3)

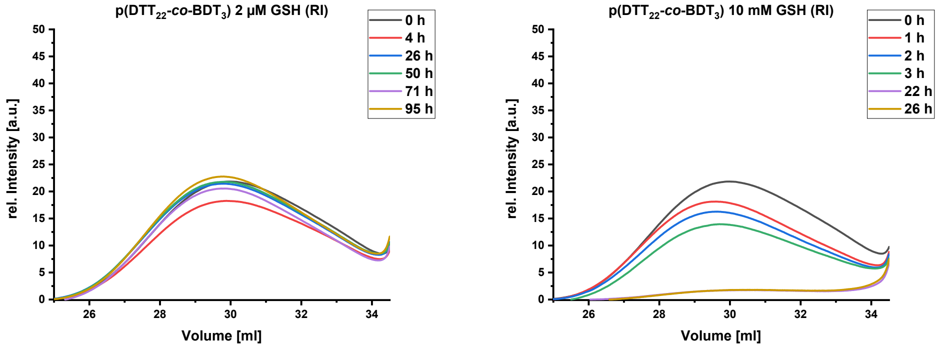

Figure S34: Degradation of p(DTT_{22}-co-BDT_3) RI Detector

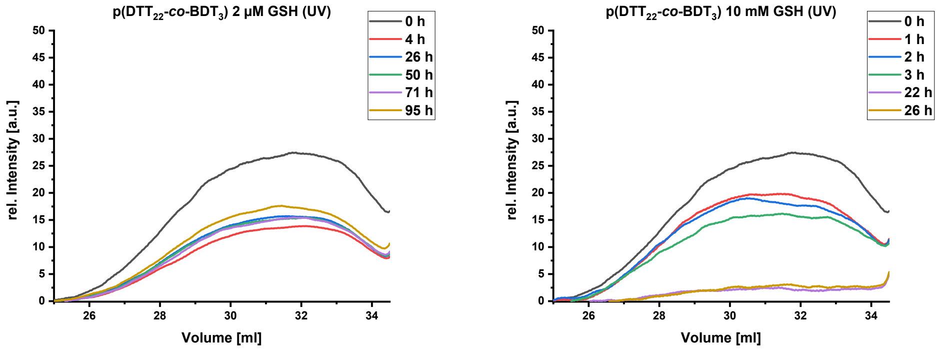

Figure S35: Degradation of p(DTT_{22}-co-BDT_3) UV Detector

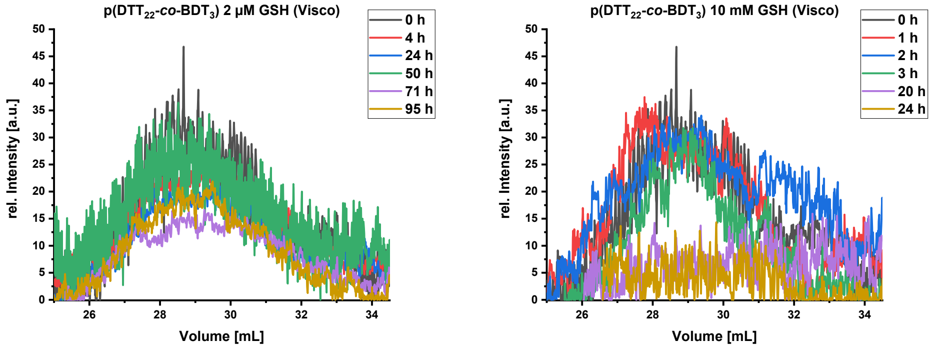

Figure S36: Degradation of p(DTT_{22}-co-BDT_3) Viscosity Detector

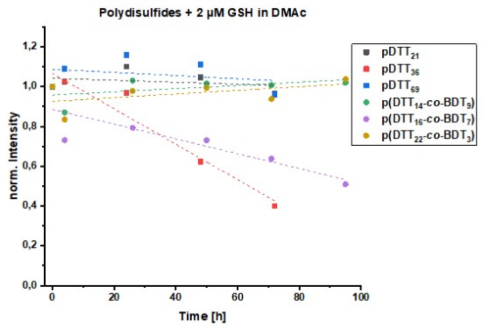

Figure S37: Degradation of homopolymers $pDTT_{21}$, $pDTT_{36}$, $pDTT_{69}$, copolymers p(DTT_{14}-co-BDT_9), p(DTT_{16}-co-BDT_7), p(DTT_{22}-co-BDT_3) in presence of 2 µM GSH in DMAc + 0.5 g L^{-1}

^{1}H-NMR results of Copolymer Degradation

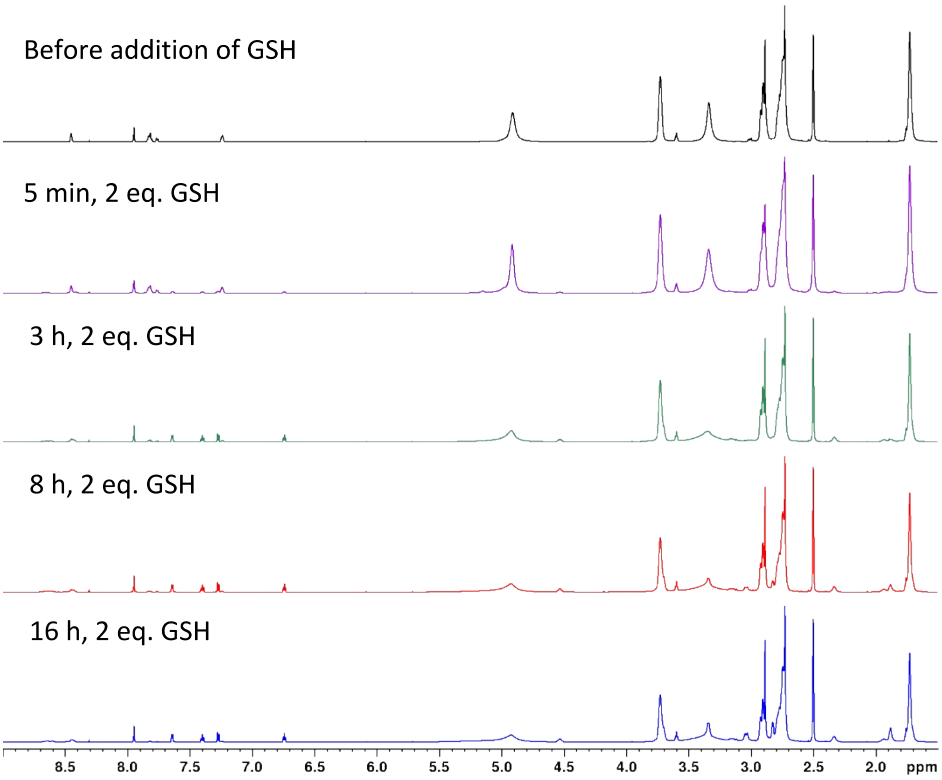

Figure S38: ^{1}H-NMR study of degradation of p(DTT_{14}-co-BDT_9) by 2 eq. GSH, spectra recorded after 5 min, 3 h, 8 h and 16 h

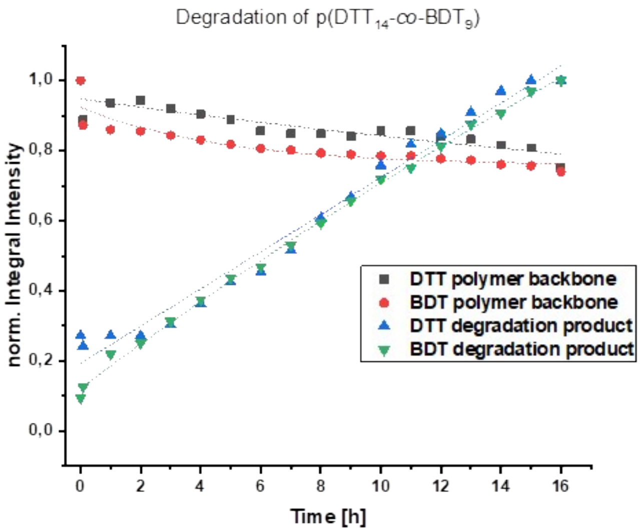

Figure S39: ^{1}H-NMR study of degradation of p(DTT_{14}-co-BDT_9) by 2 eq. GSH. Integral Intensities normalized to maximum value before addition of GSH (backbone) and after 16 h (degradation product), spectra recorded 5 min after addition, then hourly

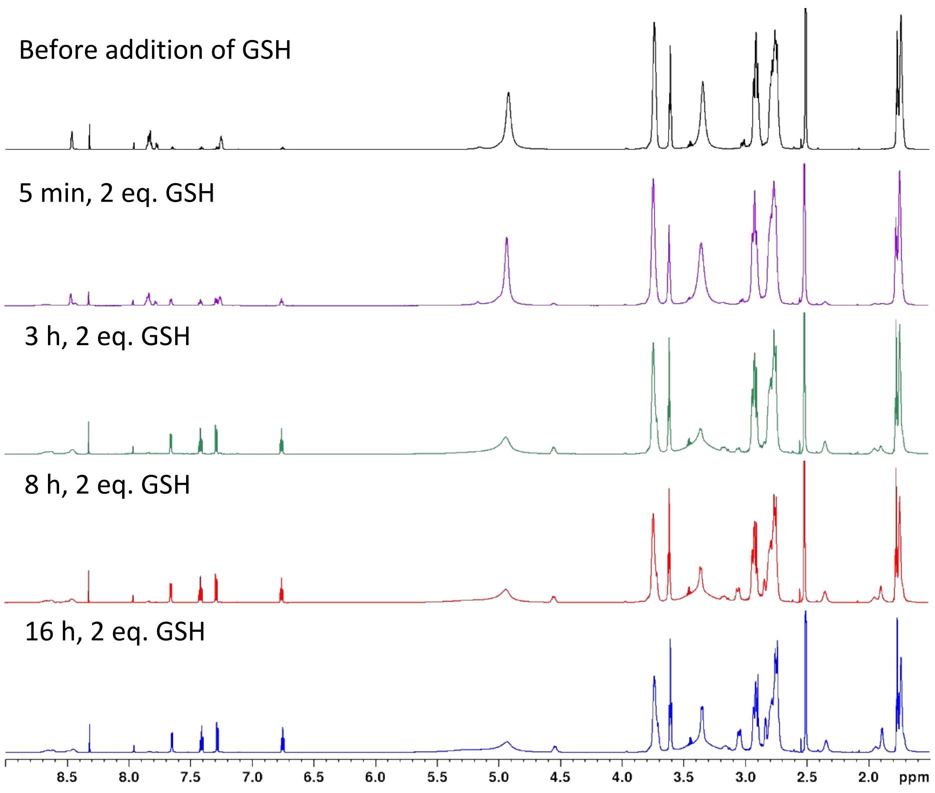

Figure S40: ^{1}H-NMR study of degradation of p(DTT_{16}-co-BDT_7) by 2 eq. GSH, spectra recorded after 5 min, 3 h, 8 h and 16 h

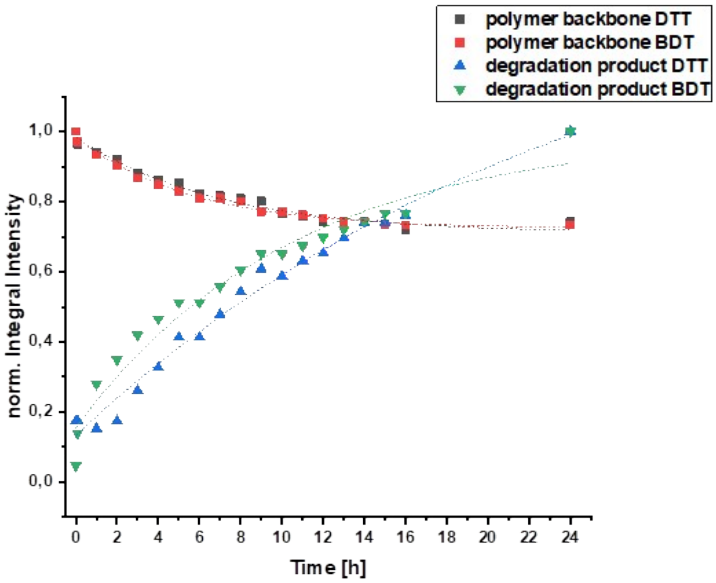

Figure S41: ^{1}H-NMR study of degradation of p(DTT_{16}-co-BDT_7) by 2 eq. GSH. Integral Intensities normalized to maximum value before addition of GSH (backbone) and after 16 h (degradation product), spectra recorded 5 min after addition, then hourly

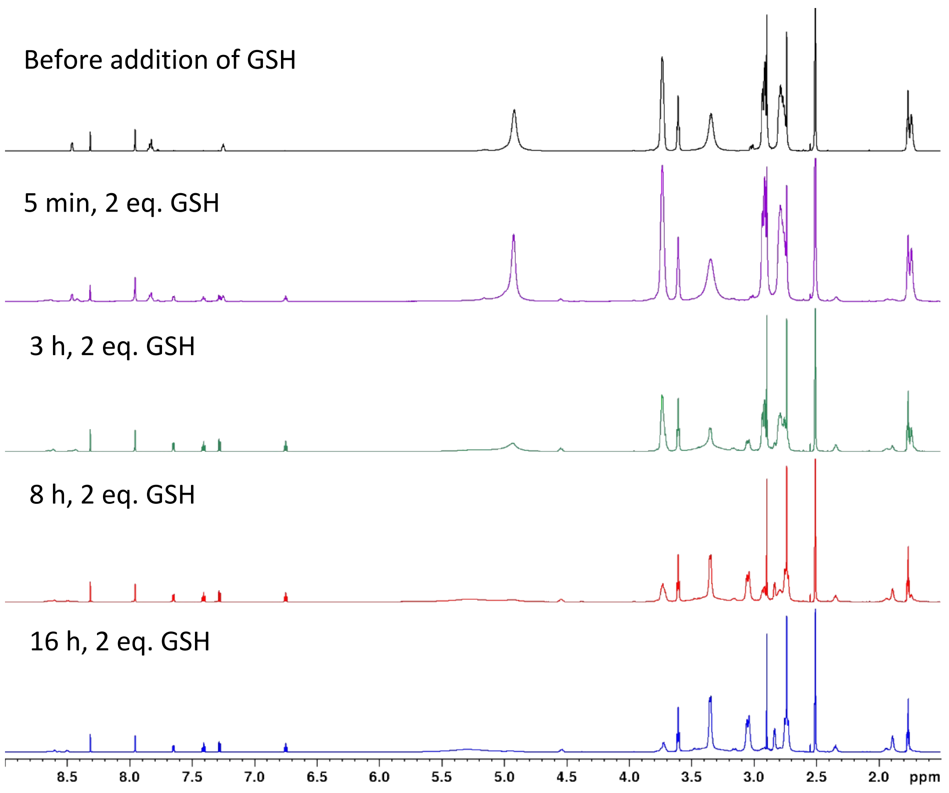

Figure S42: ¹H-NMR study of degradation of p(DTT$_{22}$-co-BDT$_3$) by 2 eq. GSH, spectra recorded after 5 min, 3 h, 8 h and 16 h

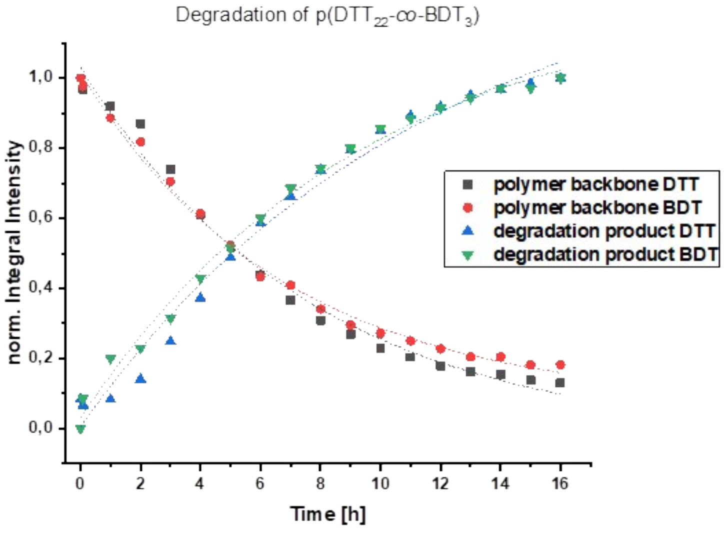

Figure S43: ¹H-NMR study of degradation of p(DTT$_{22}$-co-BDT$_3$) by 2 eq. GSH. Integral Intensities normalized to maximum value before addition of GSH (backbone) and after 16 h (degradation product), spectra recorded 5 min after addition, then hourly

Particle Degradation

Blank Particles

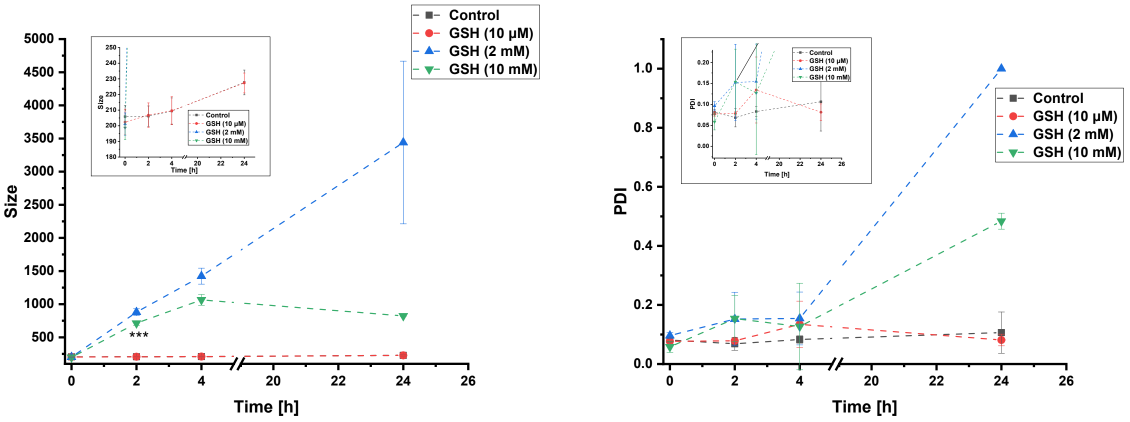

*Figure S44: DLS size and PDI of pDTT$_{36}$ particles during incubation with different GSH (mean ± SD; n = 3). Significant differences in relative count rate between physiological and pathological conditions were labelled (***$p \leq 0.001$).*

Nile Red particles

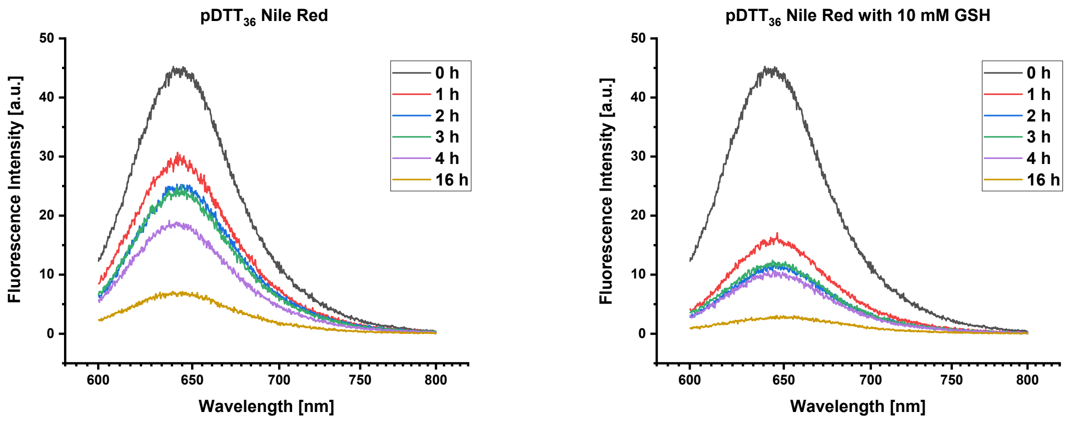

Figure S45: Fluorescence intensity of pDTT$_{36}$-Nile Red particles without and with 10 mM GSH

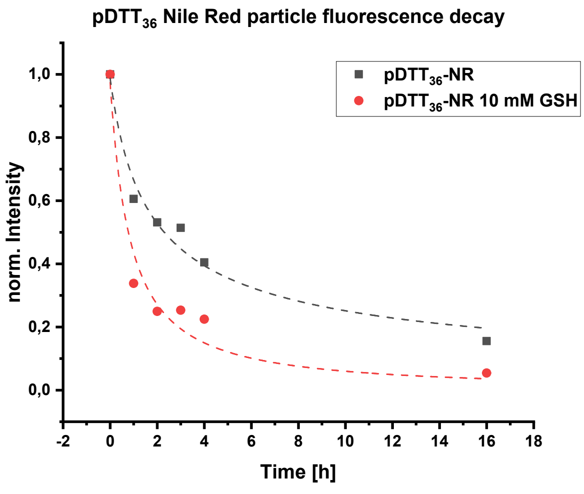

Figure S46: Normalized fluorescence intensity of pDTT$_{36}$ Nile Red (NR) particles without and with 10 mM GSH at λ_{max}=639 nm

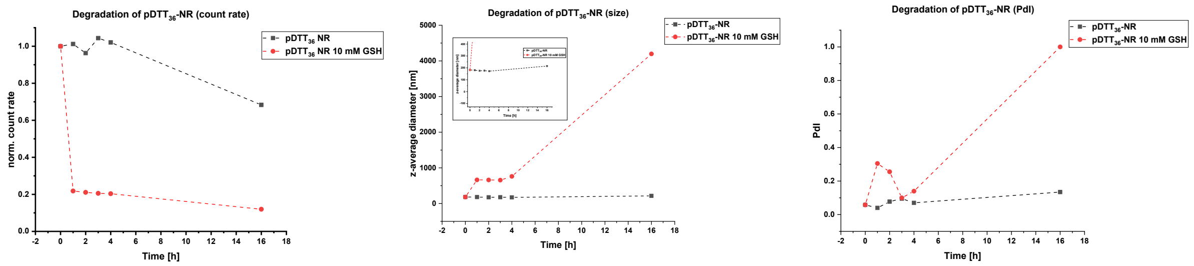

Figure S47: Normalized count rate, z-average diameter and polydispersity index of $pDTT_{36}$ Nile Red (NR) particles without and with 10 mM GSH

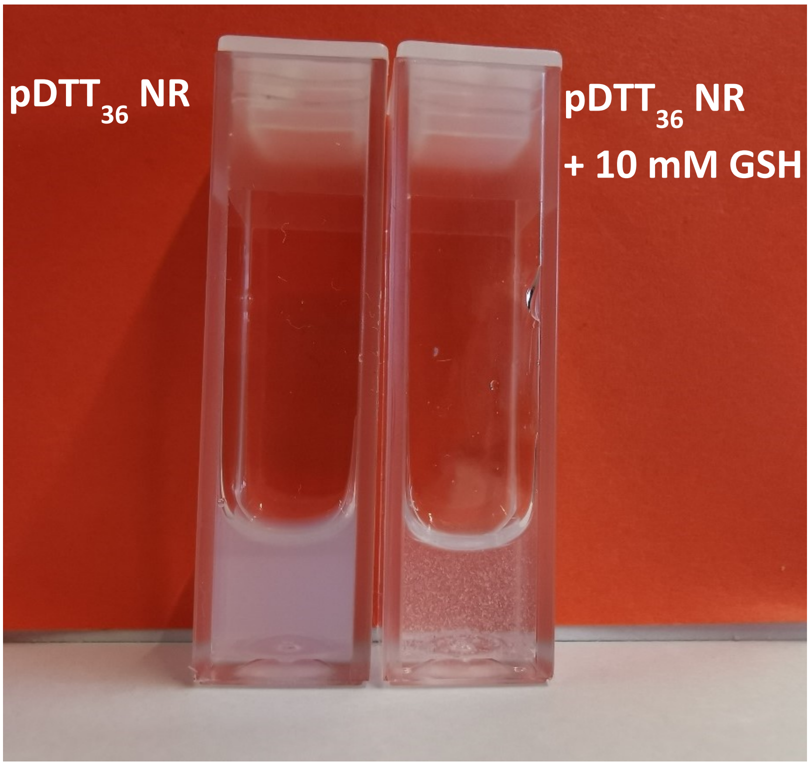

Figure S48: Undiluted $pDTT_{36}$ Nile Red (NR) particles stirred overnight at r.t. without and with 10 mM GSH

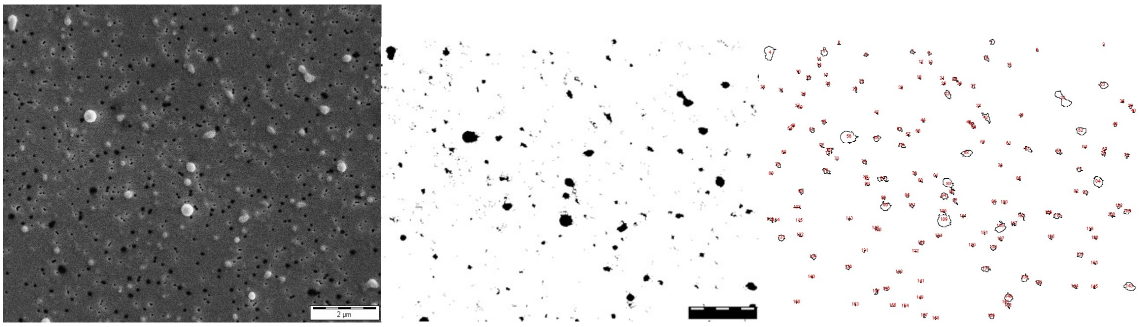

Figure S49: SEM image of $pDTT_{36}$ particles (left), grayscale extraction of particles (centre), automated particle counting by ImageJ (right)

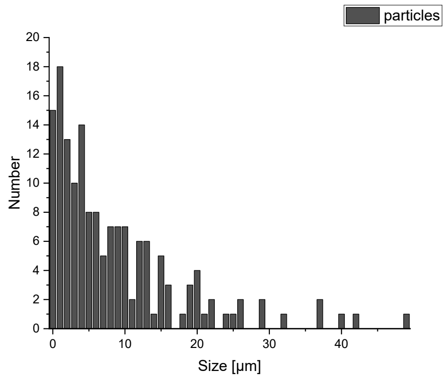

Figure S50: Size distribution of automated particle count by ImageJ (Figure S49)

Table S2: Results of automated particle count by Imagej

No.	Area [µm²]	Perimeter [µm]	Feret diameter [µm]	%Area	FeretX	FeretY	FeretAngle	MinFeret
1	0,017	0,611	0,191	100	469	14	55,158	0,16
2	0,004	0,339	0,122	100	161	9	120,823	0,091
3	0,001	0,164	0,072	100	699	14	102,612	0,031
4	0,108	1,497	0,51	100	19	18	97,037	0,346
5	0,04	0,869	0,307	100	129	38	75,252	0,219
6	0,003	0,195	0,084	100	563	25	123,871	0,047
7	0,011	0,478	0,175	100	306	29	168,517	0,12
8	0,007	0,367	0,14	100	342	30	141,407	0,103
9	0,009	0,406	0,16	100	212	37	12,597	0,105
10	0,018	0,612	0,244	100	458	37	120,823	0,143
11	0,002	0,244	0,094	100	121	43	131,829	0,07
12	0,001	0,141	0,061	100	329	50	120,823	0,043
13	0,005	0,266	0,1	100	345	53	20,427	0,087
14	0,005	0,328	0,126	100	120	54	104,344	0,084
15	0,008	0,326	0,131	100	506	54	110,985	0,094
16	0,002	0,267	0,106	100	79	68	98,484	0,053
17	0,002	0,187	0,084	100	134	76	123,871	0,054
18	0,011	0,482	0,175	100	96	86	36,682	0,122
19	0,004	0,254	0,099	100	325	80	118,236	0,075
20	0,01	0,808	0,224	100	393	81	128,851	0,149
21	0,002	0,185	0,072	100	373	82	102,612	0,047
22	0,013	0,423	0,154	100	206	94	52,507	0,125
23	0,047	0,876	0,305	100	689	95	166,763	0,244
24	0,005	0,337	0,153	100	138	91	114,109	0,057
25	0,002	0,187	0,084	100	374	97	56,129	0,045
26	0,002	0,232	0,099	100	406	98	61,764	0,047
27	0,005	0,32	0,141	100	432	97	131,829	0,078
28	0,003	0,242	0,093	100	8	105	70,303	0,062
29	0,008	0,488	0,215	100	195	100	102,612	0,069

30	0,002	0,185	0,072	100	289	101	102,612	0,047
31	0,004	0,298	0,122	100	44	105	120,823	0,063
32	0,035	0,834	0,333	100	380	107	109,191	0,173
33	0,118	1,846	0,665	100	602	110	134,84	0,314
34	0,004	0,267	0,115	100	89	113	114,109	0,062
35	0,003	0,219	0,082	100	735	132	39,963	0,069
36	0,007	0,398	0,141	100	752	144	48,171	0,113
37	0,002	0,197	0,072	100	78	138	102,612	0,058
38	0,002	0,162	0,072	100	444	142	29,19	0,044
39	0,001	0,162	0,072	100	82	142	150,81	0,046
40	0,002	0,277	0,109	100	758	153	73,388	0,062
41	0,002	0,22	0,089	100	240	151	100,148	0,047
42	0,039	1,159	0,458	100	451	153	123,06	0,194
43	0,004	0,3	0,117	100	530	162	48,171	0,075
44	0,002	0,242	0,107	100	331	167	125,603	0,049
45	0,011	0,507	0,179	100	127	168	150,81	0,134
46	0,002	0,269	0,095	100	423	171	169,451	0,07
47	0,006	0,451	0,164	100	431	173	106,612	0,109
48	0,007	0,37	0,143	100	722	173	102,612	0,086
49	0,002	0,197	0,072	100	69	178	102,612	0,058
50	0,002	0,228	0,094	100	434	185	48,171	0,059
51	0,012	0,46	0,177	100	283	183	128,065	0,128
52	0,061	0,956	0,336	100	644	184	152,11	0,262
53	0,015	0,517	0,177	100	103	185	128,065	0,141
54	0,002	0,181	0,07	100	62	185	131,829	0,052
55	0,001	0,164	0,077	100	322	189	114,109	0,031
56	0,163	1,713	0,569	100	165	206	8,821	0,426
57	0,003	0,244	0,094	100	302	196	131,829	0,07
58	0,031	0,722	0,251	100	231	203	176,005	0,198
59	0,001	0,164	0,077	100	453	215	65,891	0,031
60	0,02	0,683	0,257	100	284	214	142,277	0,15
61	0,003	0,207	0,084	100	506	218	56,129	0,062
62	0,007	0,48	0,191	100	125	215	124,842	0,087
63	0,025	0,794	0,292	100	537	224	155,294	0,158
64	0,001	0,15	0,061	100	660	222	120,823	0,047
65	0,019	0,73	0,234	100	137	225	131,829	0,192
66	0,025	0,698	0,251	100	601	228	150,81	0,175
67	0,002	0,24	0,117	100	699	230	36,682	0,043
68	0,057	1,041	0,381	100	409	236	10,549	0,244
69	0,002	0,197	0,077	100	50	232	114,109	0,06
70	0,009	0,448	0,168	100	696	240	56,129	0,114
71	0,003	0,254	0,094	100	138	238	33,838	0,07
72	0,011	0,583	0,185	100	743	235	109,697	0,133
73	0,001	0,129	0,061	100	157	246	120,823	0,031
74	0,014	0,505	0,183	100	211	249	120,823	0,139
75	0,017	0,515	0,206	100	36	254	122,067	0,141
76	0,002	0,207	0,077	100	490	263	65,891	0,06
77	0,015	0,554	0,2	100	644	266	159,573	0,142

78	0,023	0,93	0,314	100	130	265	138,208	0,177
79	0,004	0,279	0,109	100	315	274	106,612	0,076
80	0,002	0,172	0,082	100	23	276	140,037	0,042
81	0,002	0,198	0,093	100	358	279	109,697	0,031
82	0,004	0,289	0,109	100	215	287	73,388	0,072
83	0,033	1,153	0,365	100	240	284	160,429	0,207
84	0,082	1,141	0,421	100	682	282	107,292	0,297
85	0,002	0,254	0,099	100	527	285	118,236	0,067
86	0,079	1,474	0,556	100	375	286	128,224	0,291
87	0,001	0,156	0,072	100	218	289	29,19	0,035
88	0,002	0,263	0,117	100	326	289	131,829	0,054
89	0,005	0,433	0,141	100	217	295	131,829	0,105
90	0,017	0,553	0,219	100	85	319	73,388	0,125
91	0,005	0,446	0,183	100	389	310	120,823	0,082
92	0,002	0,197	0,082	100	642	311	140,037	0,055
93	0,013	0,47	0,169	100	661	311	111,692	0,14
94	0,036	0,843	0,266	100	367	323	176,24	0,228
95	0,004	0,263	0,117	100	299	324	48,171	0,064
96	0,005	0,335	0,117	100	250	324	131,829	0,094
97	0,011	0,571	0,259	100	395	326	118,845	0,103
98	0,01	0,388	0,141	100	475	338	48,171	0,122
99	0,064	1,188	0,36	100	251	332	112,974	0,308
100	0,002	0,21	0,093	100	497	333	109,697	0,046
101	0,009	0,419	0,169	100	308	337	111,692	0,112
102	0,011	0,452	0,177	100	728	347	79,852	0,109
103	0,004	0,5	0,225	100	70	344	166,535	0,066
104	0,023	0,642	0,243	100	740	357	25,587	0,157
105	0,003	0,281	0,121	100	370	352	154,413	0,052
106	0,008	0,464	0,157	100	583	358	26,408	0,105
107	0,015	0,828	0,313	100	529	355	116,722	0,145
108	0,01	0,657	0,21	100	709	360	165,542	0,132
109	0,143	1,663	0,513	100	365	359	134,428	0,419
110	0,031	0,782	0,28	100	597	365	18,192	0,175
111	0,006	0,332	0,115	100	412	360	114,109	0,103
112	0,01	0,651	0,245	100	17	365	134,552	0,142
113	0,003	0,222	0,093	100	182	371	70,303	0,062
114	0,003	0,242	0,094	100	32	374	48,171	0,062
115	0,001	0,175	0,084	100	80	370	123,871	0,036
116	0,069	1,299	0,416	100	484	395	74,763	0,301
117	0,007	0,495	0,143	100	513	377	150,81	0,122
118	0,004	0,267	0,106	100	237	384	98,484	0,062
119	0,002	0,23	0,099	100	670	392	61,764	0,047
120	0,002	0,156	0,072	100	239	390	150,81	0,035
121	0,002	0,198	0,093	100	456	395	109,697	0,031
122	0,005	0,347	0,152	100	79	399	145,072	0,078
123	0,031	0,667	0,229	100	44	400	97,84	0,203
124	0,006	0,344	0,126	100	360	402	172,049	0,091
125	0,008	0,415	0,164	100	590	401	106,612	0,088

126	0,004	0,289	0,131	100	679	404	126,717	0,064
127	0,005	0,326	0,131	100	488	406	110,985	0,078
128	0,012	0,423	0,164	100	326	420	48,171	0,125
129	0,004	0,279	0,109	100	432	419	106,612	0,078
130	0,021	0,566	0,195	100	474	421	99,243	0,171
131	0,002	0,232	0,107	100	212	436	54,397	0,049
132	0,001	0,156	0,072	100	315	433	150,81	0,035
133	0,017	0,595	0,215	100	645	444	29,19	0,15
134	0,019	0,57	0,212	100	110	451	81,516	0,162
135	0,002	0,209	0,105	100	678	456	138,208	0,035
136	0,013	0,501	0,175	100	177	462	116,438	0,143
137	0,031	0,884	0,3	100	451	466	159,573	0,192
138	0,005	0,332	0,121	100	281	474	154,413	0,07
139	0,035	0,834	0,295	100	535	495	71,447	0,219
140	0,003	0,185	0,084	100	105	484	123,871	0,047
141	0,001	0,164	0,077	100	327	497	65,891	0,031
142	0,016	0,623	0,222	100	561	494	134,84	0,145
143	0,069	1,061	0,38	100	740	502	161,219	0,274
144	0,008	0,422	0,188	100	635	503	146,162	0,09
145	0,002	0,277	0,115	100	679	503	114,109	0,059
146	0,009	0,363	0,151	100	254	507	136,238	0,097
147	0,008	0,579	0,219	100	234	521	73,388	0,102
148	0,049	0,909	0,324	100	504	517	103,961	0,256
149	0,002	0,185	0,072	100	327	526	102,612	0,047
150	0,002	0,219	0,082	100	75	539	39,963	0,061
151	0,001	0,164	0,077	100	499	539	65,891	0,031
152	0,008	0,546	0,207	100	503	539	112,137	0,107
153	0,001	0,164	0,077	100	194	540	114,109	0,031
154	0,001	0,164	0,077	100	295	547	65,891	0,031
155	0,002	0,256	0,126	100	267	544	172,049	0,017
156	0,019	0,515	0,191	100	468	561	124,842	0,137
157	0,005	0,323	0,137	100	333	568	62,913	0,062
158	0,003	0,228	0,086	100	356	568	155,919	0,07
Average=	0,015	0,462	0,172	100	359,59	273,68	105,012	0,110
